# Activation of locus coeruleus-spinal cord noradrenergic neurons alleviates neuropathic pain in mice via reducing neuroinflammation from astrocytes and microglia in spinal dorsal horn

**DOI:** 10.1186/s12974-022-02489-9

**Published:** 2022-05-27

**Authors:** Juan Li, Yiyong Wei, Junli Zhou, Helin Zou, Lulin Ma, Chengxi Liu, Zhi Xiao, Xingfeng Liu, Xinran Tan, Tian Yu, Song Cao

**Affiliations:** 1 Department of Anesthesiology, Affiliated Hospital of Zunyi Medical University, 149 Dalian Street, Zunyi, 563000 Guizhou China; 2 Department of Pain Medicine, Affiliated Hospital of Zunyi Medical University, 149 Dalian Street, Zunyi, 563000 Guizhou China; 3 Guizhou Key Lab of Anesthesia and Organ Protection, Zunyi Medical University, 6 West Xuefu Street, Zunyi, 563099 Guizhou China

**Keywords:** Locus coeruleus, Norepinephrine/noradrenaline, Microglia, Astrocyte, Pain, Noradrenergic, Spinal cord, Cytokine

## Abstract

**Background:**

The noradrenergic neurons of locus coeruleus (LC) project to the spinal dorsal horn (SDH), and release norepinephrine (NE) to inhibit pain transmission. However, its effect on pathological pain and the cellular mechanism in the SDH remains unclear. This study aimed to explore the analgesic effects and the anti-neuroinflammation mechanism of LC-spinal cord noradrenergic pathway (LC:SC) in neuropathic pain (NP) mice with sciatic chronic constriction injury.

**Methods:**

The Designer Receptors Exclusively Activated by Designer Drugs (DREADD) was used to selectively activate LC:SC. Noradrenergic neuron-specific retro–adeno-associated virus was injected to the spinal cord. Pain threshold, LC and wide dynamic range (WDR) neuron firing, neuroinflammation (microglia and astrocyte activation, cytokine expression), and α2AR expression in SDH were evaluated.

**Results:**

Activation of LC:SC with DREADD increased the mechanical and thermal nociceptive thresholds and reduced the WDR neuron firing. LC:SC activation (daily, 7 days) downregulated TNF-α and IL-1β expression, upregulated IL-4 and IL-10 expression in SDH, and inhibited microglia and astrocytes activation in NP mice. Immunofluorescence double staining confirmed that LC:SC activation decreased the expression of cytokines in microglia of the SDH. In addition, the effects of LC:SC activation could be reversed by intrathecal injection of yohimbine. Immunofluorescence of SDH showed that NE receptor α2B-AR was highly expressed in microglia in CCI mice.

**Conclusion:**

These findings indicate that selective activation of LC:SC alleviates NP in mice by increasing the release of NE and reducing neuroinflammation of astrocytes and microglia in SDH.

**Supplementary Information:**

The online version contains supplementary material available at 10.1186/s12974-022-02489-9.

## Introduction

Neuropathic pain (NP) is defined as pain caused by trauma or disease of the somatosensory system [1]. The mechanisms of NP are complicated, and there is still lacking clinically effective analgesics [2]. Therefore, NP sometimes has a long-term serious impact on the quality of life in patients.

The locus coeruleus (LC) is an important noradrenergic nucleus [3]. As one of the most important pain inhibitory pathways [4, 5], the LC-spinal cord noradrenergic pathway (LC:SC) participates in the regulation of pain, including NP [6]. The LC projects descending fibers to the spinal dorsal horn (SDH) and releases norepinephrine (NE) to inhibit the ascending of pain signals. This pathway has no obvious inhibitory effect on physiological pain, but under pathological conditions, the NE released to the SDH increases, which can significantly inhibit transmission of pathological pain [7]. For example, electrical stimulation of LC effectively alleviates NP, and this effect can be reversed by intrathecal α2 receptor antagonists [8]. The α2 receptor agonist clonidine reduces the pain in NP patients and enhances the LC:SC descending pathway by activating LC neurons [9]. Of note, Hirschberg et al. found that the activation of the LC ascending and descending pathway exerted reverse effects in NP rat [6], although activating the LC:SC reduced pain and induced conditional position preference [6].

However, whether longer time (e.g., several days) activation of the LC:SC pathway is protective in NP model, and the cellular mechanism of the analgesic effect of NE in the SDH is not fully understood. In addition, how the NE released from the LC noradrenergic neurons inhibits the transmission of NP from SDH to the brain, and its specific mechanism remains to be studied [4, 10]. Studies suggest that LC:SC noradrenergic fibers may: (1) form synapses with pain-relay neurons in the SDH and activate α2 NE receptors in the postsynaptic membrane to inhibit the excitement of secondary neurons; (2) combine with α1 receptors on inhibitory interneurons to promote the release of GABA or glycine to inhibit secondary neurons; (3) through the presynaptic mechanism: NE acts in a non-synaptic structure to act on the α2A receptors at the central end of primary pain afferent neurons in a diffuse manner, inhibiting the release of excitatory transmitters by primary neurons; (4) act on α2 receptors on excitatory interneurons in a diffuse manner, inhibiting the release of excitatory neurotransmitters and activating secondary neurons [4].

It has been confirmed that the NE receptors exist on both astrocytes and microglia derived from the brain [11, 12], and the SDH [13]. For example, NE inhibited microglia activation and cytokine expression in vitro [14]. NE also reduces the production of MCP-1 and CX3CL1 in astrocytes induced by lipopolysaccharide [15]. NE can increase the intracellular Ca^2+^ of astrocytes in the SDH mainly through α1A-AR [16]. However, it has not been reported that whether NE can act on receptors on microglia cells in the SDH in vivo.

Indirect evidence indicates that NE in the SDH may relieve NP by reducing neuroinflammation from microglia [14, 17] and astrocyte. NP has been increasingly recognized as neuroimmune diseases [18, 19]. Astrocytes and microglia with immune cell characteristics can release various anti-inflammatory (such as IL-10, IL-4, TGF-β) and pro-inflammatory (such as BDNF, TNF-α, IL-1β) cytokines [20, 21]. These glial cell-derived inflammatory cytokines promote the occurrence, development, and maintenance of chronic pain, including NP. In vivo (brain) and in vitro (brain-derived glial cells) experiments have confirmed that NE acts on glial cells to exert significant anti-inflammatory effect [17]. The degenerative changes of LC (LC neuron and projection fiber loss) are accompanied by elevated microglial neuroinflammation in mouse brain and spinal cord [22]. If NE derived from LC is released into the SDH in a diffuse manner, it may act on NE receptors on microglia and astrocytes to inhibit these cells from producing inflammatory mediators, thereby alleviating NP.

Although it has been reported that selectively activating the LC:SC noradrenergic pathway can increase the pain threshold in NP rat [6], it is unclear whether LC:SC activation relieves NP and reduced neuroinflammation is not clear, and the specific cellular and molecular mechanisms are still unknown. In the present study, we hypothesize that activating the LC:SC can increase NE release and alleviate NP by inhibiting neuroinflammation from activated microglia and astrocyte in the SDH.

## Materials and methods

### Animals

This study was approved by the Animal Care and Use Committees of Zunyi Medical University (KLLY(A)-2019-021). Animal studies were performed in accordance with the Guide for the Care and Use of Laboratory Animals [23]. In the present study, only males were selected to control variables. C57/BL6J wild-type mice (8–12 weeks old, 25–30 g) were purchased from the Changsha Tianqin Biotechnology (Changsha, China). Three to four mice were accommodated in a cage at a constant room temperature of 23 ± 2 °C and relative humidity of 55% ± 2% with a 12-h light/dark circle. The food and water were freely accessed. All mice adapted to the environment for 1 week before experiments. The sciatic chronic constriction injury (CCI) mouse was used as the NP model [24].

### Viral vectors

The adeno-associated virus (AAV), i.e., the rAAV2-retro gene delivery system can be used in combination with the Cre recombinase driver system to achieve long-term, high-level transgene expression, which is sufficient for functional inquiry of neural circuit functions and genome editing in target neuronal populations [25].

AAV viruses encoding Ef1a-DIO-hM3Dq-mCherry and retrograde AAV viruses encoding tyrosine hydroxylase (TH)-NLS-CRE were ordered from BrainVTA (Wuhan, China). The virus titer is 3–8 × 10^12^ genome copies/ml, and the virus vector is divided into aliquots and stored at − 80 °C [26].

### Virus injection

The mice were anesthetized with 3% sevoflurane and put into a stereotaxic device (RWD, Shenzhen, China). Erythromycin ointment was used to prevent corneal dryness, and a heat pad (RWD, Shenzhen, China) was used to keep the body temperature at 37 °C. A dental drill (OmniDrill 35, WPI, USA), a typical stereotactic syringe (Stoelting, Wood Dale, IL, USA), and a controller (Micro4, WPI, Sarasota, USA) connected to the micropipette were used to make a small craniotomy hole for injection.

To selectively target the LC:SC pathway, 200 nl virus solution (rAAV-TH-NLS-CRE-WPRE-hGH-pA, AAV2/retro) was injected into the SDH (L3-L5), and another 200 nl virus solution (Efla-DIO-hM3Dq-mCherry or Efla-DIO-mCherry) into the LC (AP: -5.4 mm, ML: ± 1.0 mm, DV: -3.5 mm). Specifically, the vertebral column was exposed and clamped at vertebral segment L3. A laminectomy was performed at T12-L1 and 200 nl virus solution was injected into the L3–L5 segments of the side ipsilateral to the nerve constriction. This injection was located 0.5 mm lateral from the midline and 0.3 mm deep from the dorsal surface of the spinal cord [27]. The pipette was held in place for 10 min, and then withdrawn slowly. After the injection, the wound was sutured, antibiotics (penicillin) and carprofen were applied to the surgical wound, and the animal was resuscitated under a heating lamp. The analgesia lasted for about 24 h and did not affect the follow-up experiments [28].

### Immunofluorescence

The mice were anesthetized with urethane (1.7 g/kg, i.p.), perfused with phosphate buffer saline (PBS), and then fixed with 4% paraformaldehyde (PFA). The spinal cords and brains were taken immediately for frozen sectioning. Then the tissue was transferred to 30% sucrose to dehydrate after the overnight of post-fixing with 4% PFA. After sunk to the bottom, the tissue was cut into 25 μm thickness with freezing microtome (Leica, Wetzlar, Germany). Sections were washed using PBS with 0.3% Triton X-100 (5 min, 3 times), then blocked with 1% BSA and 0.3% Triton X-100 in the PBS for 2 h. Then, sections were incubated with rabbit anti-IBA1 (1:1000; Wako, Osaka, Japan), rabbit anti-TH (1:1000; Abcam, Cambridge, UK), mouse anti-TNF-α (1:1000; Abcam, Cambridge, UK), mouse anti-IL-1β (1:1000; Santa Cruz, Dallas, USA), goat anti-α2A-AR (1:50, PAB6968, Abnova), mouse anti-α2B-AR (1:50, Santa Cruz, Shanghai, China), rabbit anti-NeuN (1:1000, Cell Signaling, Massachusetts, USA), and rabbit anti-GFAP (1:1000; Abcam, Cambridge, UK) antibody, respectively, and refrigerated at 4 °C overnight. After washing the primary antibody with PBS containing 0.3% Triton X-100 (5 min, 3 times), sections were added with secondary antibody, goat anti-rabbit IgG H&L (1:1000; Abcam, Cambridge, UK) and goat anti-mouse IgG H&L (1:1000; Abcam, Cambridge, UK), and incubated for 2 h at room temperature without light. Finally, sections were attached on glass slide, photographed under a fluorescence microscope (Olympus, Tokyo, Japan). Image J (v.1.8 NIH, Washington, USA) were used to calculate cell fluorescence proportion.

To quantify IBA1, GFAP, IL-1β, and TNF-α expression, the spinal cord (L3-L5) images were outlined with the size-standardized regions of interest (ROIs) in the SDH [22] by the ImageJ software (v.1.8), and the percentage of fluorescence-positive area was quantified with ImageJ. The total number of cells in each ROI is counted with the aid of ImageJ, and the double-positive cells (yellow fluorescence) are manually judged and counted [22] by a lab technician who does not know the experimental design and grouping.

### In vivo extracellular recordings from the LC and intraperitoneal injection of CNO

Anesthesia was induced with 3% sevoflurane to achieve loss of paw withdrawal reflex. The mouse was placed in a stereotaxic frame, the skull exposed, and the screw above the viral injection site removed to gain access to the brain. In vivo units were recorded with a single channel electrode (Monopolar Tengsten Electrode, WE3003X, Microprobes for Life Science, USA) connected to a multi-channel fiber photometry system (Thinkertech, Nanjing, China). Neuronal activity was recorded via the multi-channel fiber photometry system, and Spike2 software (CED, Cambridge Electronic Design, UK) was used to store data. Action potential discharge frequency was compared before and 60 min after CNO application.

### Enzyme-linked immunosorbent assay (ELISA)

Mice were anesthetized and perfused with PBS; then the lumbar and sacral spinal cord segments were collected and stored at − 80 °C. Before ELISA experiment, spinal cord tissue was homogenized in 0.1 M PBS with protease inhibitor cocktail (20 μl/100 mg tissue, P8340, Sigma, Israel), and centrifuged at 1000 rpm, 4 °C for 10 min. Finally, the supernatant was collected; then the total protein content was determined with BCA assay kit (23,225, Thermo Fisher, USA). The concentration of the supernatant was normalized to the protein content, and the levels of TNF-α, IL-1β (Beyotime, Shanghai, China), and NE (Elabscience, Wuhan, China) were detected with ELISA kits following the manufacturers' instructions. Optical density of each well at 450 nm was measured.

### CCI model

When the mice were anesthetized with 3% sevoflurane, the sciatic nerve was bluntly separated, and the left sciatic nerve was lightly ligated with 5–0 silk thread. The tightness of the ligation is that the mouse leg muscles will tremble slightly during the ligation, and the distance between 4 ligations is about 1 mm. In the sham operation group, except that the sciatic nerve was not ligated, the other operations were the same as those in the CCI group.

### Intrathecal injection

Intrathecal injection follows the procedure described below. In brief, a 10 μl microsyringe with a tube and needle was inserted through the skin into the L5/L6 intervertebral space of the subarachnoid space [29, 30]. Sudden tail flick after insertion was considered a sign of successful injection. A volume of 5 μl of the test drug was injected into the subarachnoid space via a microsyringe.

### Drug preparation and administration

Clozapine N-oxide (CNO, MCE, New Jersey, USA) and yohimbine (MCE, New Jersey, USA) are both diluted to 1 mg/ml with 0.9% NaCl. Three weeks after virus injection and CCI model establishment, mice were randomly divided into eight groups: Sham, CCI, CCI + Vector + Saline, CCI + Vector + CNO, CCI + M3 + Saline, CCI + M3 + CNO, CCI + M3 + CNO + Saline, and CCI + M3 + CNO + Yohimbine group. Then the CCI + M3 + CNO and CCI + Vector + CNO mice were intraperitoneally injected with a dose of 3 mg/kg CNO with a syringe, while the CCI + M3 + saline and CCI + Vector + Saline mice received the same amount of saline. CCI + M3 + CNO + Yohimbine mice were injected with CNO [31] and intrathecal injected 5 μl yohimbine [32] between the L4/L5 intervertebral space with an insulin syringe (BD, USA) [33]. CCI + M3 + CNO + Saline mice received the same amount of saline. All injection reagents were injected once a day for 7 consecutive days, and the pain thresholds were measured 3 days after the last injection.

### Mechanical pain threshold assessment

Mice were placed on a mesh suspension plate and adapted to the environment for 30 min. Mechanical withdrawal thresholds were tested by a von Frey filament kit (Danmic Global, USA) with the up-down method [34–36].

### Thermal nociceptive threshold assessment

Mice were individually placed in a plexiglass enclosure on a transparent glass surface maintained at 25 °C. After adapting to the environment for 30 min, thermal nociceptive thresholds were detected with a plantar radiant heat pain tester (IITC, Wood Dale, IL, USA), which consisted of a light-emitting projection lamp and an electronic timer. After placing the light beam under the left hind paw vertically, the device will be activated. The time it recorded automatically before the mouse lift their hind paw was exactly the thermal withdrawal latency. The cut-off time, which was used to prevent damage to the left foot of mice, was set as 30 s. Each mouse was performed 5 times with a 5 min interval. The average of the five tests was considered as the thermal withdrawal latency.

### In vivo extracellular recordings of wide dynamic range neurons in the SDH

Animals were anesthetized with 3% sevoflurane. The spinal cord lumbar enlargement (L3-L5) was exposed by laminectomy for recordings [37]. Neuronal activity was detected with a multi-channel electrode (2601, Thinkertech Nanjing Bioscience Inc., China) and amplified using a multi-channel fiber photometry system. The data were stored using Spike2 software. Wide dynamic range (WDR) neurons were identified in the deep dorsal horn by graded responses to both non-noxious touch and noxious pinch. Their discharge frequency was measured in response to light touch with a brush or to sustained pinch pressure over five second time bins immediately after stimulus application.

### Quantitative real-time polymerase chain reaction (qRT-PCR)

The dorsal parts of spinal cords were collected [24] and used to extract the total RNA with RNAiso Plus (TaKaRa, Shiga, Japan). Then PrimeScript™ RT reagent kit with gDNA Eraser (TaKaRa, Shiga, Japan) was used to erase genomic DNA and synthesized cDNA in the total RNA. The qRT-PCR was performed using CFX96 Real-Time PCR Detection System (Bio-Rad, California, USA) with TB Green™ Premix Ex Taq™ II (TaKaRa, Shiga, Japan) at 95 °C for 30 s and 40 cycles of 95 °C for 5 s followed by 60 °C for 30 s. The primer sequences are listed in Table 1. The data were calculated with 2^−ΔΔCt^ method.

**Table 1:** Primer sequences, related to Fig. 6

**Genes**		**Primer sequences**
TNF-α	F	5’-AGCAGAAGCTCCCTCAGCGAGG- 3’
	R	5’-TCCACGTCGCGGATCATGCTTT-3’
IL-1β	F	5’-CTGCTTCCAAACCTTTGACCTG-3’
	R	5’-TGGGCTCTTCTTCAAAGATGAA-3’
CD68	F	5’-ACAGGCAGCACAGTGGACATTCA-3’
	R	5’-AGAAACATGGCCCGAAGTGTCCC-3’
iNOS	F	5’-TGAGGGCTGTAGCCCTATTTCA-3’
	R	5’-CTGTGGACGGGTCGATGTCACA-3’
IL-6	F	5’-GCTCTGGAGCCCACCAAGAACG-3’
	R	5’-CCTCCGACTTGTGAAGTGGTAT-3’
CD11b	F	5’-GTTTTTACCCCTCCCTCCTGGC-3’
	R	5’-TCTGTCCAAAGCCTTTTGCATT-3’
CD206	F	5’-GACCTTGGACTGAGCAAAGGGG-3’
	R	5’-AGAGCGTCCACGCAGCGCTTGT-3’
IL-4	F	5’-TTGTTAGCATCTCTTGATAAAC-3’
	R	5’-GCATGGCGTCCCTTCTCCTGTG-3’
Arg1	F	5’-GTGCCCTCTGTCTTTTAGGGTTA-3’
	R	5’-GTTTTTCCAGCAGACCAGCTTTC-3’
IL-10	F	5’-TTAGAGACTTGCTCTTGCACTA-3’
	R	5’-GGCTGAAGGCAGTCCGCAGCTC-3’
GAPDH	F	5’-GCTCATGACCACAGTCCATGC-3’
	R	5’-CAGATCCACGACGGACACATTG-3’

*F* forward, *R* reverse

### Statistical analysis

Graphpad Prism 8.0 (GraphPad Software, La Jolla, USA) and SPSS 19.0 (IBM, Chicago, IL, USA) were used for statistical analysis. Data are expressed as mean ± SEM. One-way analysis of variance was used for comparisons between multiple groups at the same time point. Two-way repeated measures analysis of variance was used for comparisons before and after the same group at different time points. The Tukey's test was used for multiple comparisons between two groups. Immunofluorescence images were analyzed statistically using Image J (v.1.8).

## Results

### Chemogenetic activation of LC:SC neurons

**Fig. 1:**
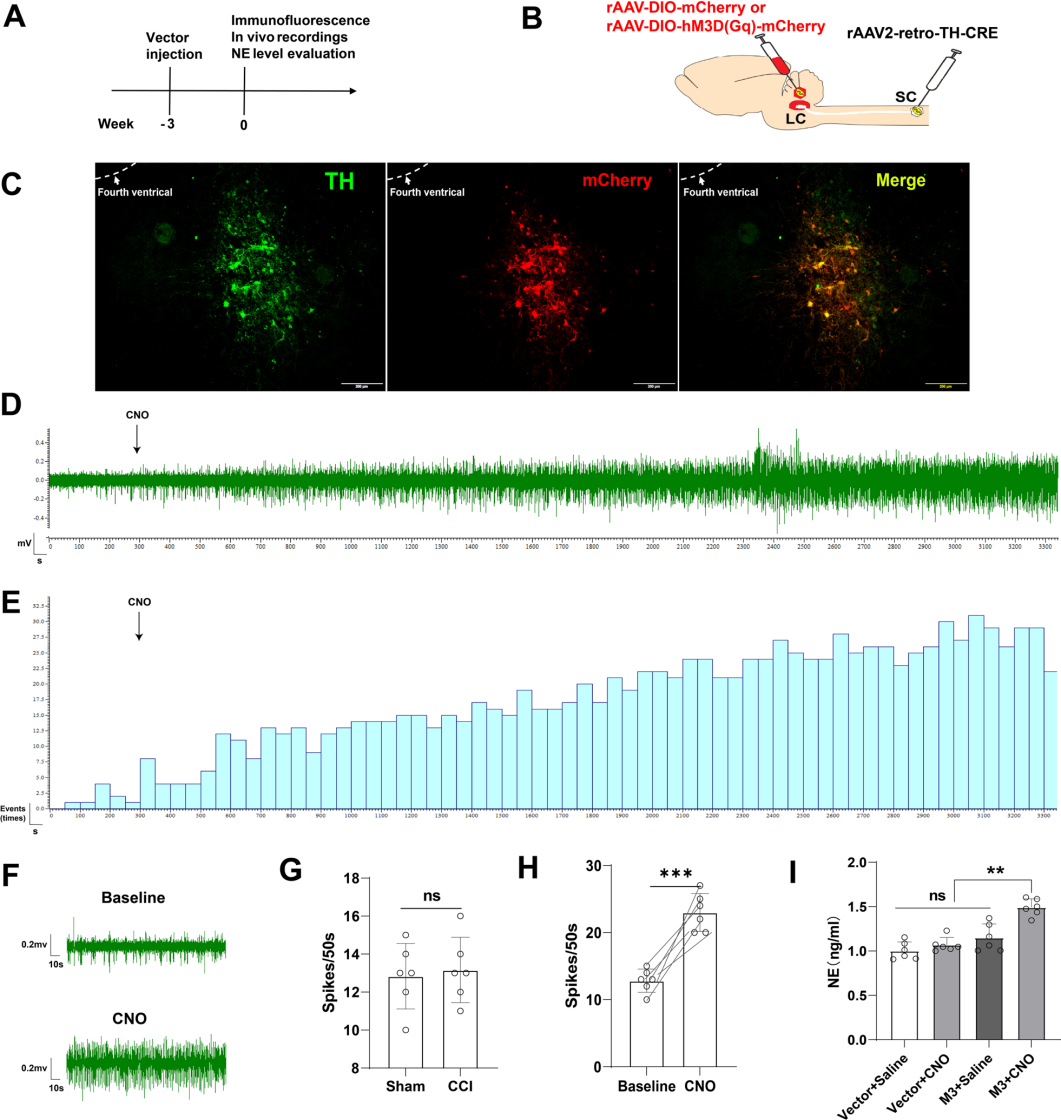
Chemogenetic activation of LC neurons in vivo. **A** Experimental protocol for vector injection, immunofluorescence, and in vivo extracellular recordings in the LC. **B** Schematic representation of the Gq-DREADD protocol targeting LC neurons. **C** Expression of M3-mCherry in LC and its co-expression with LC noradrenergic (TH positive) neuron. Bar = 200 μm. **D** The discharge of the LC neurons recorded using a single electrode placed in the LC. **E** The firing frequency distribution diagram of neurons in the LC. **F** Changes of LC neuron firing before and 60 min after peritoneal injection of CNO. **G** Baseline firing frequency of LC neurons in Sham and CCI mice. **H** Intraperitoneal injection of CNO increased the firing frequency of LC neurons. **I** NE contents in SDH increased after LC:SC activation with Gq-DREADD. Data are expressed as mean + SEM, *n* = 6 for electrophysiological recordings and ELISA tests. **p < 0.01, ***p < 0.001. *ns* not statistically significant

In order to selectively activate the LC:SC pathway, we used Gq-DREADD (Gq-coupled receptor, hM3Dq) and its ligand CNO. In order to enable the overexpression of the Gq-DREADD receptor in LC TH expression neurons, the virus (TH-NLS-CRE AAV2/retro, Efla-DIO-hM3Dq-mCherry, or Efla-DIO-mCherry) was injected into the SDH (L3-L5) and the LC, respectively, while the intraperitoneal injection of CNO guaranteed the selective activation of the LC:SC pathway. Three weeks after virus injection, the M3 expression in the LC neurons was detected. The in vivo extracellular recordings of the spike of LC neuron will be conducted 3 weeks after surgery (Fig. 1A, B).

The immunofluorescence of slices showed that the red (M3-mCherry) and the green (TH-positive LC neurons) overlapped in LC after microinjection of chemogenetic virus into the SDH and LC 3 weeks later. Almost only TH-positive neurons can express mCherry. The overlap between the two is more than 10% in all the TH-positive neurons, and most of the mCherry-expressing neurons are located at the ventral part of LC. In the caudal part of LC, more TH-positive neurons can be transfected by retrograde AAV (Fig. 1C). The results suggested that noradrenergic neurons in the LC were successfully transfected and the M3 receptor was expressed in the noradrenergic neurons mainly in the ventral and caudal part of LC (Fig. 1C).

The mouse was fixed on the in vitro locator to locate the LC and place the metal single electrode. The electrophysiological recordings of LC were made on the side ipsilateral to the nerve injury. After recording the discharge of the LC neurons, the mice were injected with CNO into the abdominal cavity. About 5 min later, the firing frequency of the LC neurons gradually increased (Fig. 1D, E). CCI did not affect the baseline firing frequency of LC neurons (Fig. 1G). Compared with the baseline, the frequency of neuron firing in LC increased after intraperitoneal injection of CNO (Fig. 1F, H). NE contents in SDH increased after LC:SC activation with Gq-DREADD (Fig. 1I). These results indicated that the LC NE neurons can be activated by M3 Gq-DREADD.

### Activation of LC:SC relieves NP in CCI mice

**Fig. 2:**
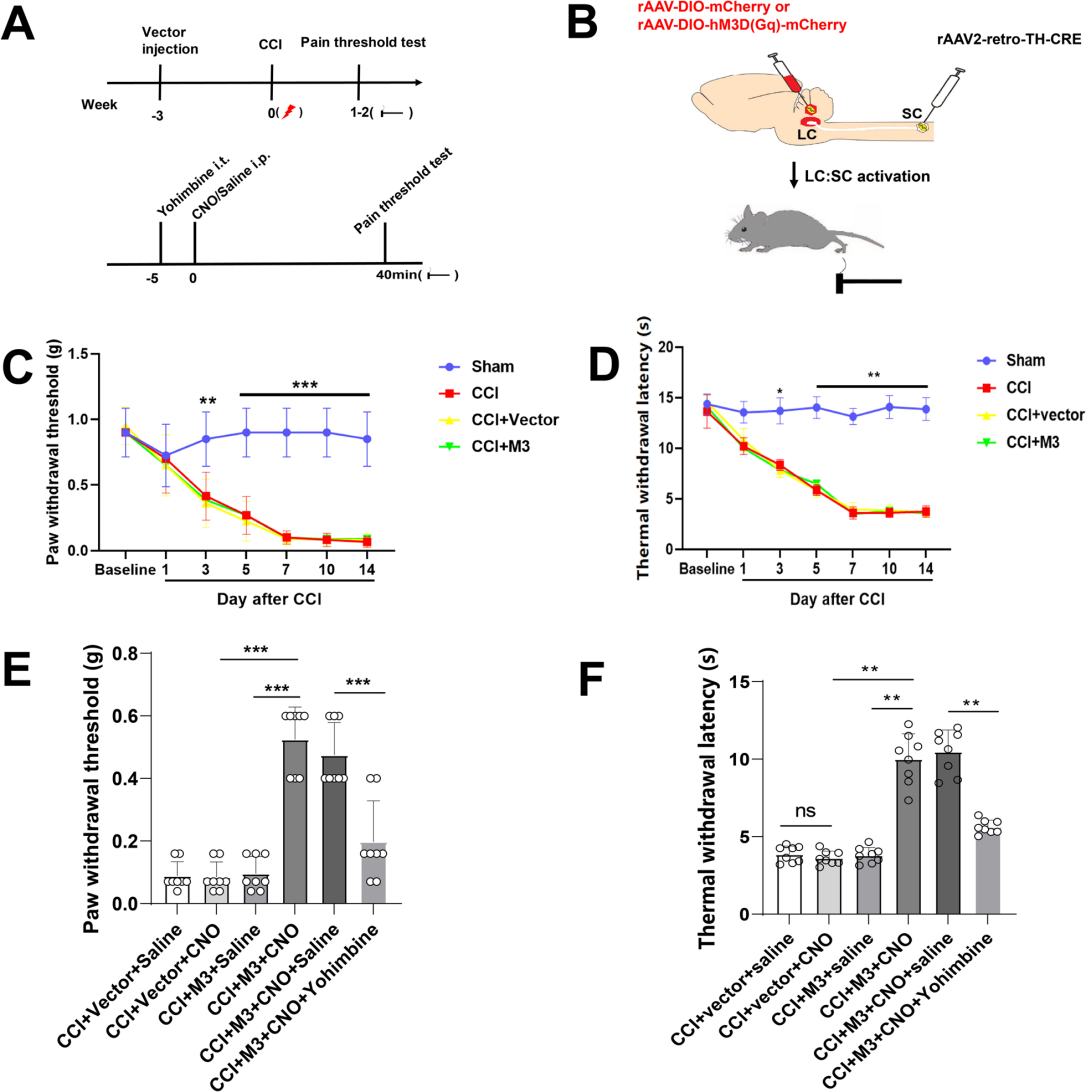
Activating the LC:SC reduces hyperalgesia in CCI mice. **A** Experimental protocol for AAV injection and pain threshold test. *i.t.* intrathecal injection. *i.p.* intraperitoneal injection. **B** Schematic representation of the Gq-DREADD protocol targeting LC neurons before pain threshold evaluation. **C** Changes of mechanical pain threshold in mice after surgery. **D** Changes of thermal nociceptive threshold in mice after surgery. **E** Paw withdrawal threshold tested with von Frey filaments decreased in CCI + M3 + Saline group, CCI + Vector + CNO + Yohimbine group, and CCI + Vector + CNO group, compared with the CCI + M3 + CNO group. **F** Thermal withdrawal latency measured by a plantar thermal testing apparatus reduced in CCI + M3 + Saline group, CCI + Vector + CNO + Yohimbine group, and CCI + Vector + CNO group, compared with the CCI + M3 + CNO group. Data are expressed as mean + SEM, *n* = 8 for mechanical withdrawal threshold tests and thermal withdrawal latency tests. **p < 0.01, ***p < 0.001, *ns* not statistically significant

The CCI model was established 3 weeks after AAV injection, and the pain threshold of mice was detected 1–2 weeks after CCI surgery (Fig. 2A, B). Compared with the sham operation mice, the mechanical withdrawal threshold of mice in the CCI group decreased significantly. The lowest pain threshold appears on the 7th day after surgery. Compared with the Sham group, the thermal withdrawal latency of mice in the CCI group dropped significantly to the lowest on the 7th day after surgery. Until 14th days after surgery, the pain threshold level was consistently low (Fig. 2C, D). These data indicated that CCI resulted in NP from the 7th day after surgery. Yohimbine was given intrathecally (3 μg/μl in sterile saline with 20% DMSO) [6] 5 min before CNO administration.

Compared with the CCI + M3 + Saline group, the mechanical pain threshold and thermal nociceptive thresholds of the CCI + M3 + CNO group significantly increased. Compared with the CCI + Vector + CNO group, the mechanical and thermal nociceptive pain thresholds of the CCI + M3 + CNO group increased. However, compared with the CCI + M3 + CNO + Saline group, the mechanical pain threshold and thermal nociceptive pain thresholds of the CCI + M3 + CNO + Yohimbine group significantly reduced (Fig. 2E, F). CNO or CNO + Yohimbine did not change the mechanical paw withdrawal thresholds and thermal withdrawal latencies of the contralateral hind paw before and after injection of CNO or CNO + Yohimbine (Additional file 1: Fig. S1). These results suggested that the LC:SC activation reduced hyperalgesia of CCI mice.

### Activation of LC:SC inhibited firing of WDR neurons in CCI mice

**Fig. 3:**
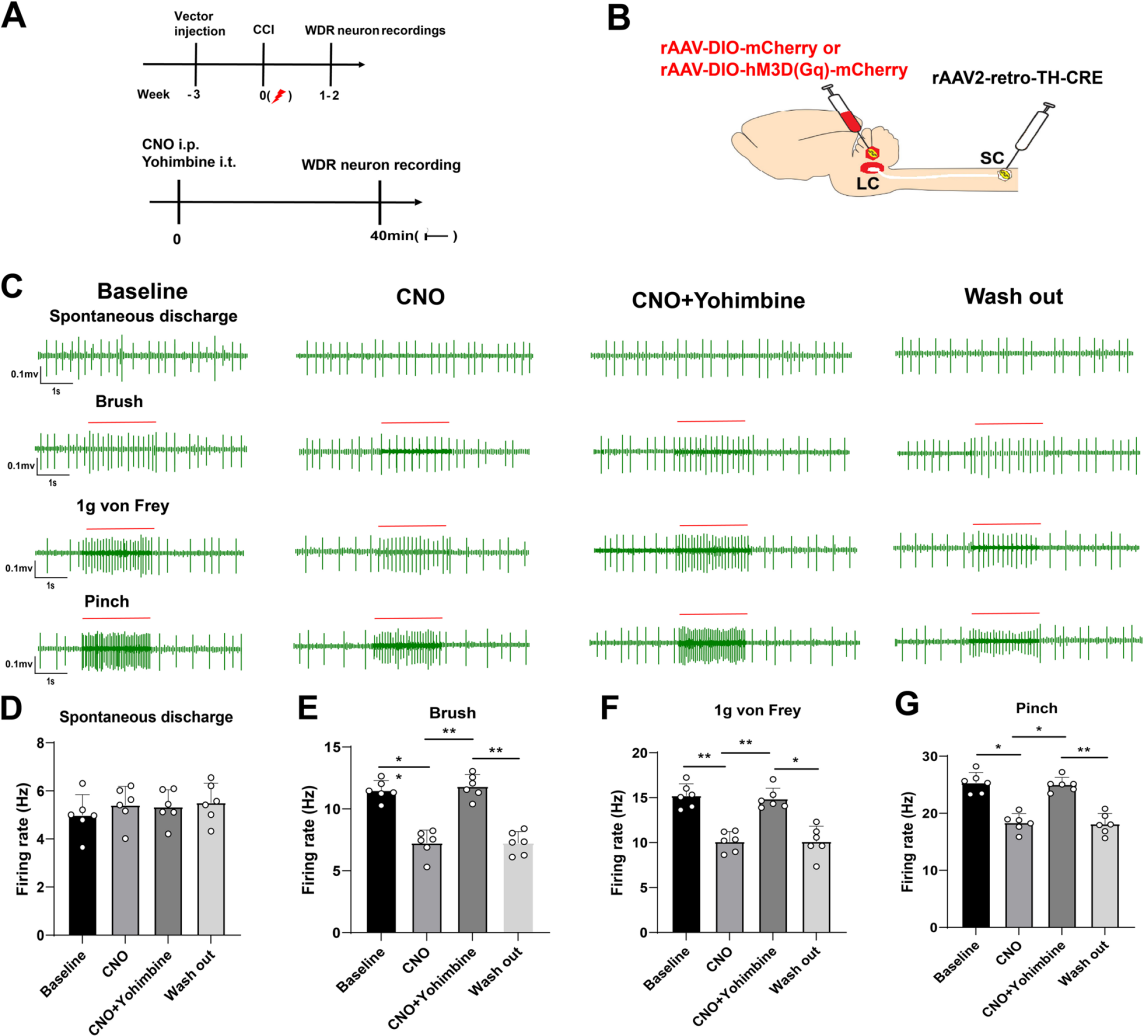
Activation of LC:SC suppresses the firing frequency of WDR neurons in CCI mice. **A** Experimental protocol for AAV injection and pain threshold test. i.p.: intraperitoneal injection. i.t.: intrathecal injection. **B** Schematic representation of the Gq-DREADD protocol targeting LC:SC. **C** Experimental protocol for noxious pinch or non-noxious brush and 1 g von Frey applied to the ipsilateral hind paw during spinal drug application. Original traces of pinch or brush and 1 g von Frey responses from WDR neurons during application of CNO (i.p., 3 mg/kg) or CNO (i.p., 3 mg/kg) + Yohimbine (i.p., 0.1 μg/kg). **D** There was no obvious change of the spontaneous discharge of the WDR neuron in CCI mice. **E** The frequency of electric discharge in the CNO group was reduced compared with the baseline value. Compared with the CNO group, the firing frequency of WDR neurons in the CNO + Yohimbine group was increased. Compared with the CNO + Yohimbine group, the firing frequency of WDR neurons in the wash-out group was lower. **F** In LC:SC activated mice, there was a significant attenuation of the response to 1 g von Frey induced by yohimbine, which indicated a spinal α2-adrenoceptor mechanism. **G** The firing frequency in the CNO group was decreased compared with the baseline, whereas it was lower in the wash-out group than that in the CNO + Yohimbine group. Data are expressed as mean + SEM, *n* = 6 mice. *p < 0.05, **p < 0.01, *ns* not statistically significant

To investigate whether chemogenetic activation of LC:SC reduces induced pain transmission, WDR neurons activity was evaluated with extracellular recordings in the SDH (mean depth 600 μm, range from 500 to 1000 μm) in a preparation that allowed the application of drugs directly onto the spinal cord in the irrigating solution. The spinal electrophysiological recordings were recorded on the side ipsilateral to the nerve injury. The baseline firing rate of LC neurons in CCI mice is not different from that of sham mice (Additional file 1: Fig. S2). The results showed that the firing frequency of WDR neurons gradually increased with the increase of stimulation in the plantar of mice (Fig. 3C).

WDR neurons encode stimulus intensity, responding to both innocuous and noxious stimuli. The average spontaneous discharge frequency of WDR was 5.08 ± 0.29 Hz, which was increased to 25.02 ± 1.18 Hz by pinch stimulation of the hind paw (Fig. 3D, G). In LC:SC transfected animals, the response of WDR units to all stimuli was reversibly attenuated by the application of CNO (3 mg/kg) and this effect was blocked by concurrent perfusion of yohimbine (0.1 μg/kg [6], Fig. 3C–G). The spontaneous firing of the WDR neurons was unaffected by CNO (Fig. 3D). These data demonstrated that the activation of LC:SC in CCI mice caused a α2-adrenoceptor-mediated inhibition of both responses to innocuous and noxious stimuli in WDR neurons.

### Activation of LC:SC relieved NP of CCI mice

To investigate whether chemogenetic activation of LC:SC can reduce NP, NE levels were detected 1 week after the activation of LC:SC, and the pain threshold of CCI mice was evaluated 3 days after LC:SC activation.

**Fig. 4:**
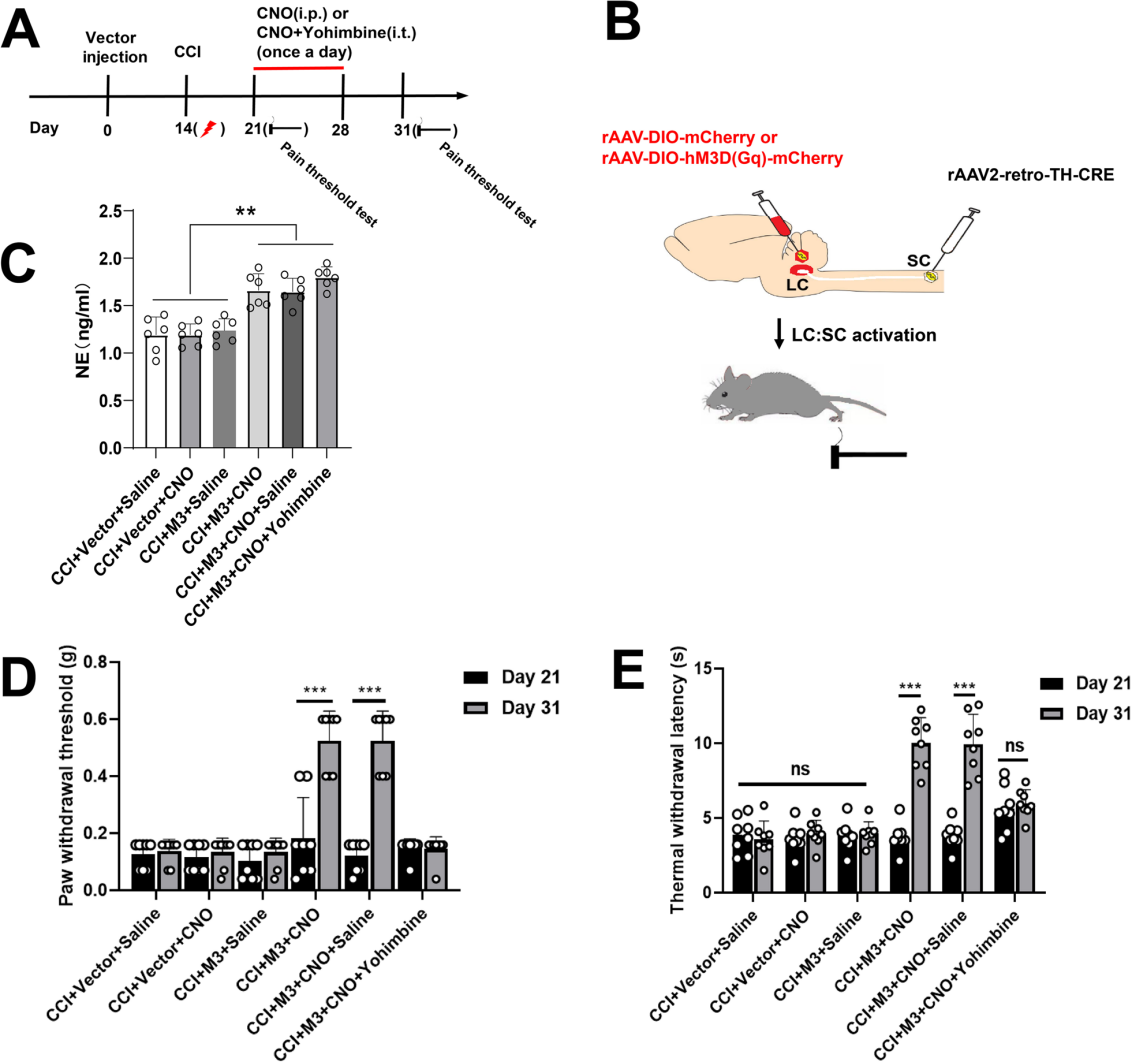
LC:SC activation for one week increases NE in SDH and relieves NP.** A** Experimental protocol for AAV injection, CCI surgery, and pain threshold test. *i.t.* intrathecal injection. *i.p.* intraperitoneal injection. **B** Schematic diagram of LC and spinal cord virus injection in mice. **C** The content of NE in the dorsal spinal cord that was measured by ELISA increased in CCI + M3 + CNO group compared with that of the CCI + M3 + Saline group. No significant differences were observed for the remaining groups. **D** On the 31st day, paw withdrawal threshold tested with von Frey filaments increased in CCI + M3 + CNO group and CCI + M3 + CNO + Saline group compared with that on the 21st day after vector injection. **E** On the 31st day, thermal withdrawal latency measured by a plantar thermal testing apparatus in CCI + M3 + CNO group and CCI + M3 + CNO + saline group compared with that on the 21st day after vector injection. Data are expressed as mean + SEM, *n* = 6 for NE content evaluation, *n* = 8 for mechanical withdrawal threshold tests and thermal withdrawal latency tests. **p < 0.01, ***p < 0.001, *ns* not statistically significant

The content of NE in the dorsal spinal cord was measured by ELISA. NE content increased in CCI + M3 + CNO group compared with the CCI + M3 + Saline group. The results showed that chemogenetic activation of LC:SC increased NE level (Fig. 4C). Long-term LC:SC activation also reduced NP three days after LC:SC activation (Fig. 4D, E). This effect was blocked by intrathecal injection of yohimbine (1 mg/ml in 5 μl [32], Fig. 4D, E). These data demonstrated that activation of LC:SC in CCI mice caused a α2-adrenoceptor-mediated inhibition of hyperalgesia.

### Activation of LC:SC for one week inhibited the activation of microglia and astrocyte in SDH

**Fig. 5:**
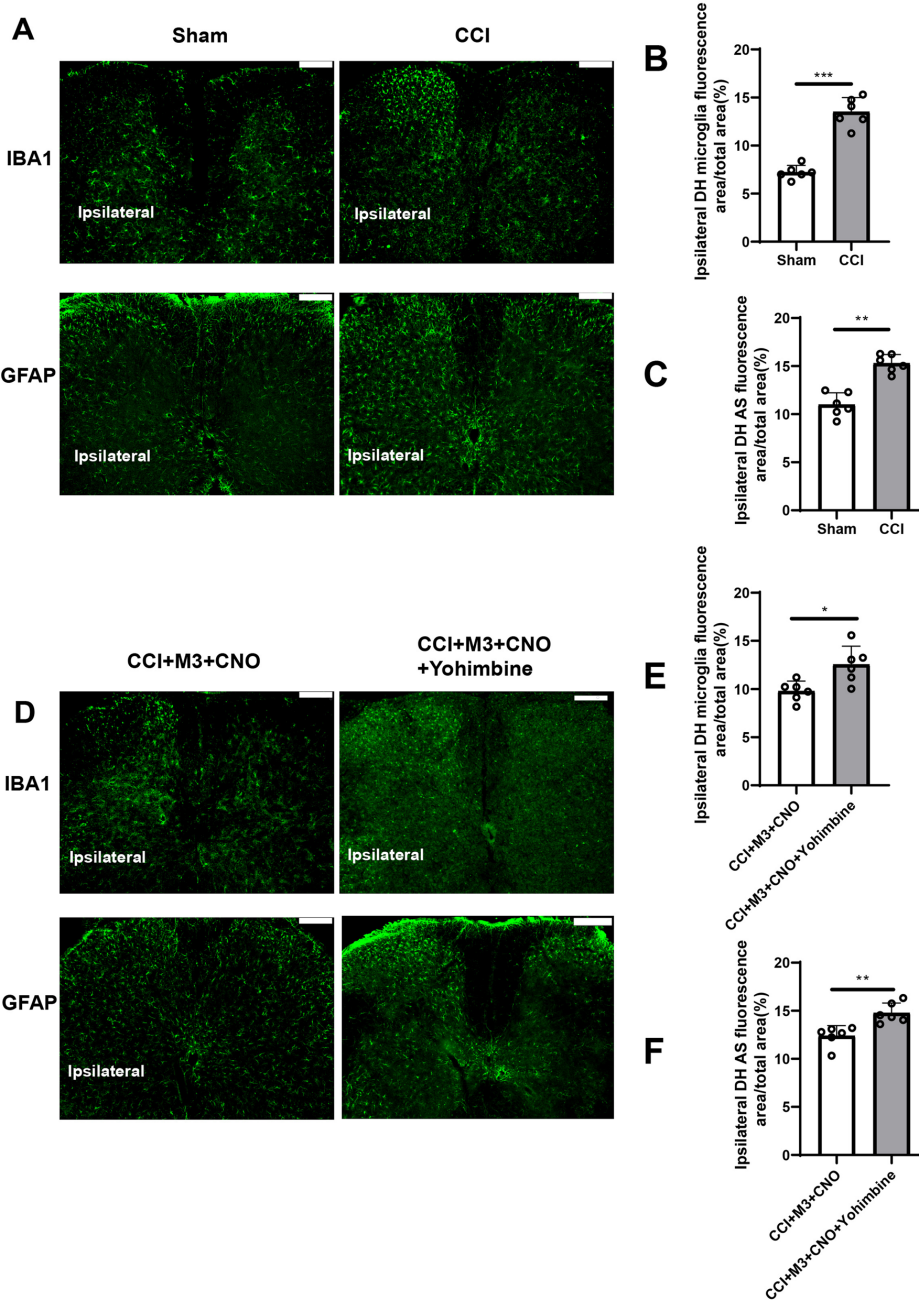
Activation of LC:SC for a week suppresses the activation of microglia and astrocyte in SDH.** A** Immunofluorescence images of microglia and astrocytes in the SDH in CCI mice. Scale bars = 100 μm. **B**, **C** Proportions of IBA1-positive area **B** and GFAP-positive area **C** in the SDH, which indicated that microglia and astrocyte (AS) were activated after CCI surgery. **D** Activation of LC:SC suppresses the activation of microglia and astrocyte in SDH. Scale bars = 100 μm. **E**, **F** Statistics of the proportion of IBA1 **E-** and GFAP **F**-positive area in the SDH, which indicated that one week of LC:SC activation can reverse the activation of astrocyte and microglia in CCI mice. Data are expressed as mean + SEM, *n* = 6, 5 slices for each mouse. *p < 0.05, **p < 0.01, ***p < 0.001. *ns* not statistically significant

Immunofluorescence was used to assess the activation of microglia and astrocyte in the SDH (L3-L5). Compared with Sham group, more microglia and astrocyte were activated in the CCI-ipsilateral SDH (Fig. 5A–C).

After one week of LC:SC activation, compared with the CCI + M3 + Saline group, the activation of astrocyte and microglia in the CCI + M3 + CNO group decreased significantly. Compared with the CCI + M3 + CNO + Saline group, the activation of astrocyte and microglia in the CCI + M3 + CNO + Yohimbine group increased (Fig. 5D–F, Additional file 1: Fig. S3).

### Activation of LC:SC inhibited neuroinflammation in SDH

**Fig. 6:**
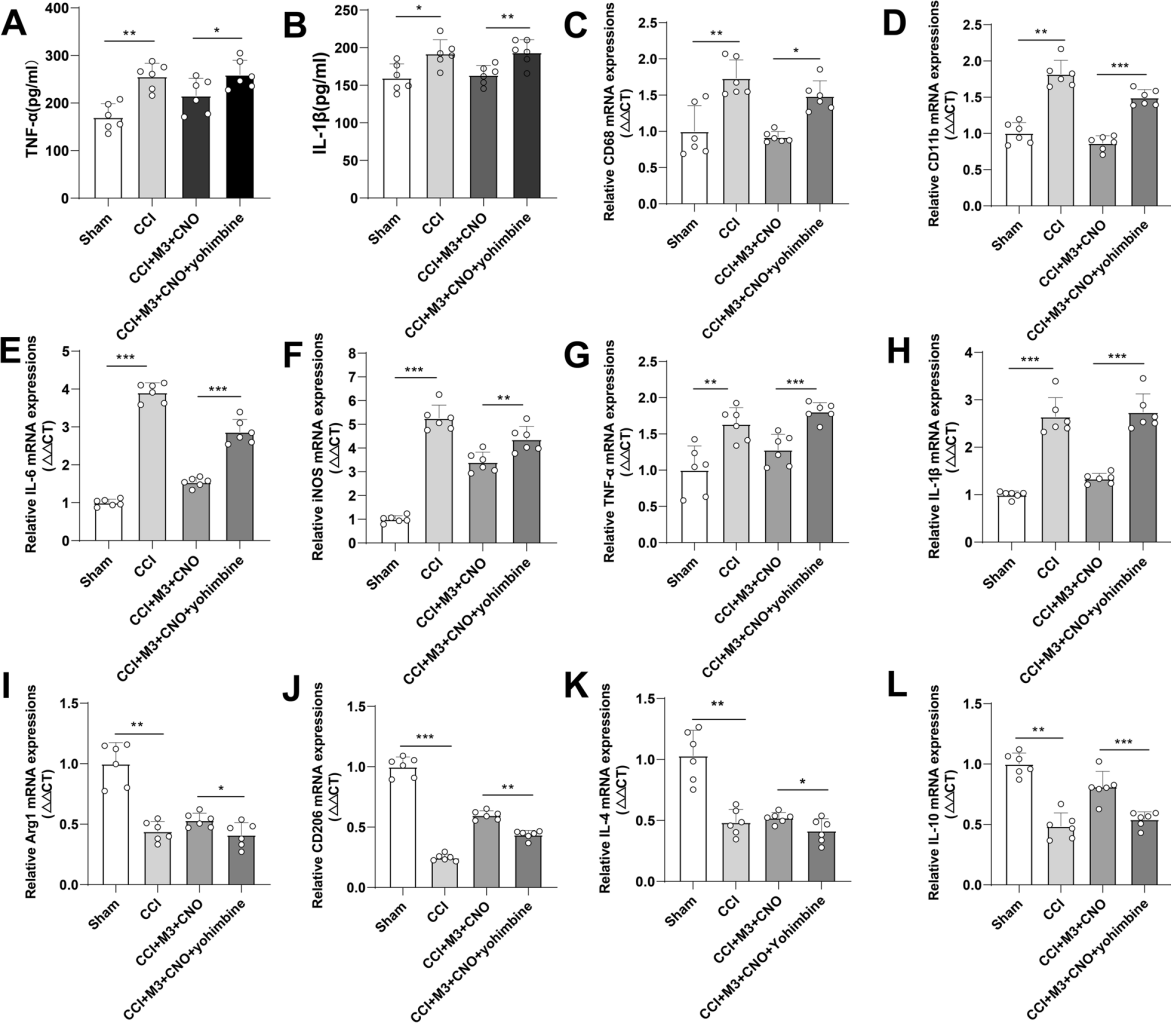
Activation of LC:SC promotes anti-inflammatory cytokines and inhibits pro-inflammatory cytokines in SDH.** A** CCI increased TNF-α expression in SDH, whereas activation of LC:SC inhibited the expression of TNF-α. **B** CCI increased IL-1β expression in SDH, whereas activation of LC:SC inhibited the expression of IL-1β. **C**–**H** CCI increased the mRNA expression of TNF-α, IL-1β, CD68, IL-6, iNOS, and CD11b, whereas activation of LC:SC inhibited the expression of these mRNAs in the SDH of CCI mice. Yohimbine reversed LC:SC activation induced downregulation of these pro-inflammation mRNAs. **I**–**L** CCI inhibited the mRNA expression of Arg1, CD206, IL-4, and IL-10, whereas activation of LC:SC increased the expression of these mRNAs in the SDH of CCI mice. Yohimbine reversed LC:SC induced upregulation of these anti-inflammation mRNAs. Data are expressed as mean + SEM, *n* = 6. Statistical analyses consisted of one-way ANOVA tests followed by Tukey’s post hoc tests. *p < 0.05, **p < 0.01, ***p < 0.001. ns = not statistically significant

Considering that LC:SC activation suppressed microglia and astrocyte activation, to evaluate the anti-neuroinflammation effects of LC:SC, we examined several glia-related cytokines after long-term (7 days) LC:SC activation. Compared with the Sham group, the expressions of TNF-α and IL-1β in the SDH of CCI group upregulated. Compared with the CCI + M3 + Saline group, in the CCI + M3 + CNO group, the expressions of TNF-α and IL-1β downregulated (Fig. 6A, B, Additional file 1: Fig. S4). The PCR was used to detect the neuroinflammation of the SDH. The results showed that compared with Sham group, the expression of pro-inflammatory cytokine mRNA in CCI group upregulated. Compared with the CCI + Vector + CNO group, the expression of pro-inflammatory cytokines in CCI + M3 + CNO group downregulated significantly. Compared with the CCI + M3 + Saline group, the expression of pro-inflammatory cytokines in CCI + M3 + CNO group decreased. In addition, compared with CCI + M3 + CNO + Saline group, the expression of pro-inflammatory cytokines in CCI + M3 + CNO + Yohimbine group was reversed by yohimbine (Fig. 6C–H, Additional file 1: Fig. S4).

Compared with Sham group, in CCI group, the expression of anti-inflammatory cytokines in the SDH decreased. Compared with the CCI + Vector + CNO group, the expression of pro-inflammatory cytokines decreased in the SDH in CCI + M3 + CNO group. Compared with the CCI + M3 + Saline group, the expression of pro-inflammatory cytokines in the CCI + M3 + CNO group decreased. In addition, in CCI + M3 + CNO + Yohimbine group, compared with CCI + M3 + CNO group, the expression of anti-inflammatory cytokines decreased (Fig. 6I–L, Additional file 1: Fig. S4).

**Fig. 7:**
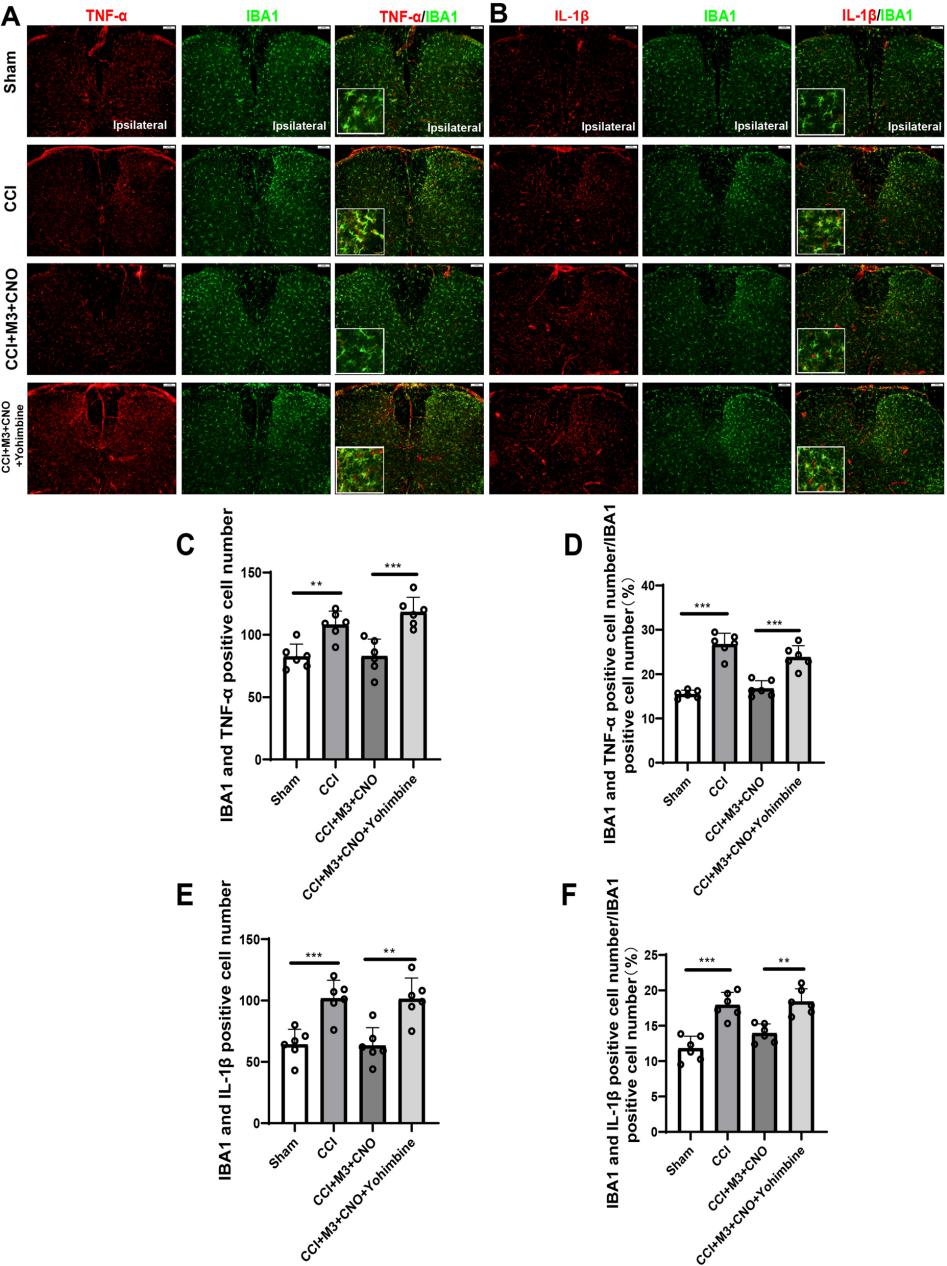
Co-expression of TNF-α, IL-1β with IBA1 in the SDH.** A** Colocalization of TNF-α (red) and IBA1 (green) in the SDH following CCI. More colocalized microglia (yellow) can be seen in mice with CCI. **B** The representative images of double immunofluorescence staining showing that IL-1β (red) colocalized with IBA1 (green) in SDH. **C**, **D** Shown in relative number and in percentage, colocalization of TNF-α and IBA1 in CCI group increased compared to the Sham group. Compared with the CCI group, colocalization of TNF-α and IBA1 of the CCI + M3 + CNO group is reduced and can be reversed by yohimbine. **E**, **F** Colocalization of IL-1β and IBA1 in CCI group increased compared to the Sham group. Compared with the CCI group, colocalization of IL-1β and IBA1 of the CCI + M3 + CNO group is reduced and can be reversed by yohimbine. Scale bars = 100 μm. Data are expressed as mean + SEM, *n* = 6, 5 slices for each mouse. *p < 0.05, **p < 0.01, ***p < 0.001

In order to determine if microglia or astrocytes expressed the aforementioned cytokines in the spinal cord (L3-L5), immunofluorescence was used to observe the co-labeling of inflammatory cytokines and glial cell markers (IBA1 or GFAP). The results showed that both TNF-α and IL-1β were positively correlated with the number and activation of microglia (Fig. 7A, B, Additional file 1: Fig. S5). The relative number and percentage of colocalization of TNF-α and IBA1 in CCI group increased compared to these of the Sham group. Compared with the CCI + M3 + Saline group, colocalization of TNF-α and IBA1 of the CCI + M3 + CNO group reduced and can be reversed by yohimbine (Fig. 7C, Additional file 1: Fig. S5). Compared with the Sham group, the colocalization of IL-1β and IBA1 in the CCI group increased in relative numbers and percentages. Compared with the CCI + M3 + Saline group, the colocalization of IL-1β and IBA1 in the CCI + M3 + CNO group reduced, which could be reversed by yohimbine (Fig. 7E, F, Additional file 1: Fig. S5).

**Fig. 8:**
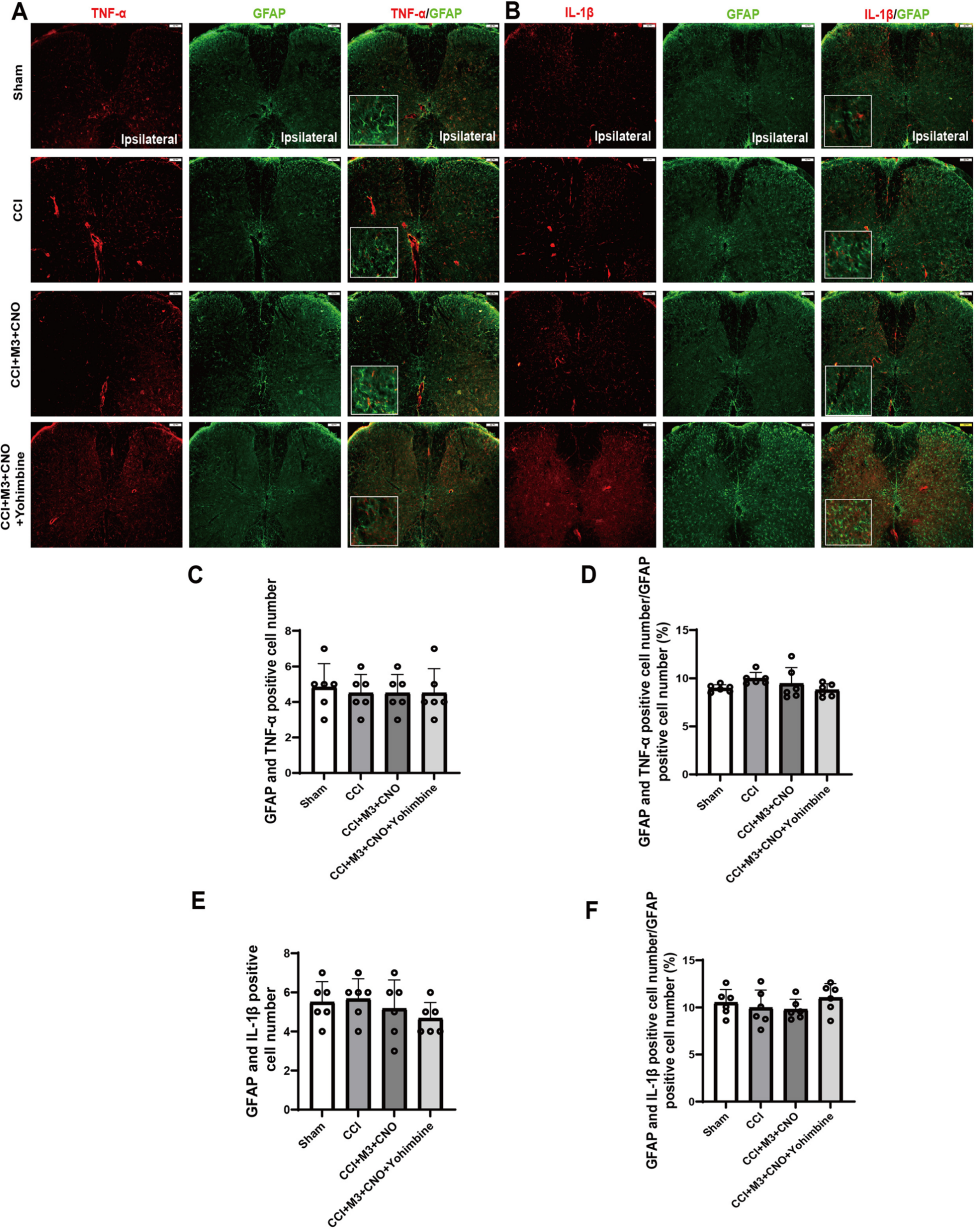
Co-expression of TNF-α, IL-1β with GFAP in the SDH. **A** Colocalization of TNF-α (red) and GFAP (green) in the SDH following CCI. Not too much colocalized astrocytes (yellow) can be seen in mice with CCI. **B** The representative images of double immunofluorescence staining showing that IL-1β (red) is not well colocalized with GFAP (green) in SDH. **C**, **D** Shown in relative number and in percentage, colocalization of TNF-α and GFAP in groups was comparable. **E**, **F** Shown in relative number and in percentage, colocalization of IL-1β and GFAP in groups was comparable. Scale bars = 100 μm. Data are expressed as mean + SEM, *n* = 6, 5 slices for each mouse

However, both TNF-α and IL-1β could not well co-expressed with astrocytes, indicating that inflammatory cytokines of spinal cord after CCI were not mainly produced by astrocytes (Fig. 8, Additional file 1: Fig. S6).

### α2-AR expression in SDH

**Fig. 9:**
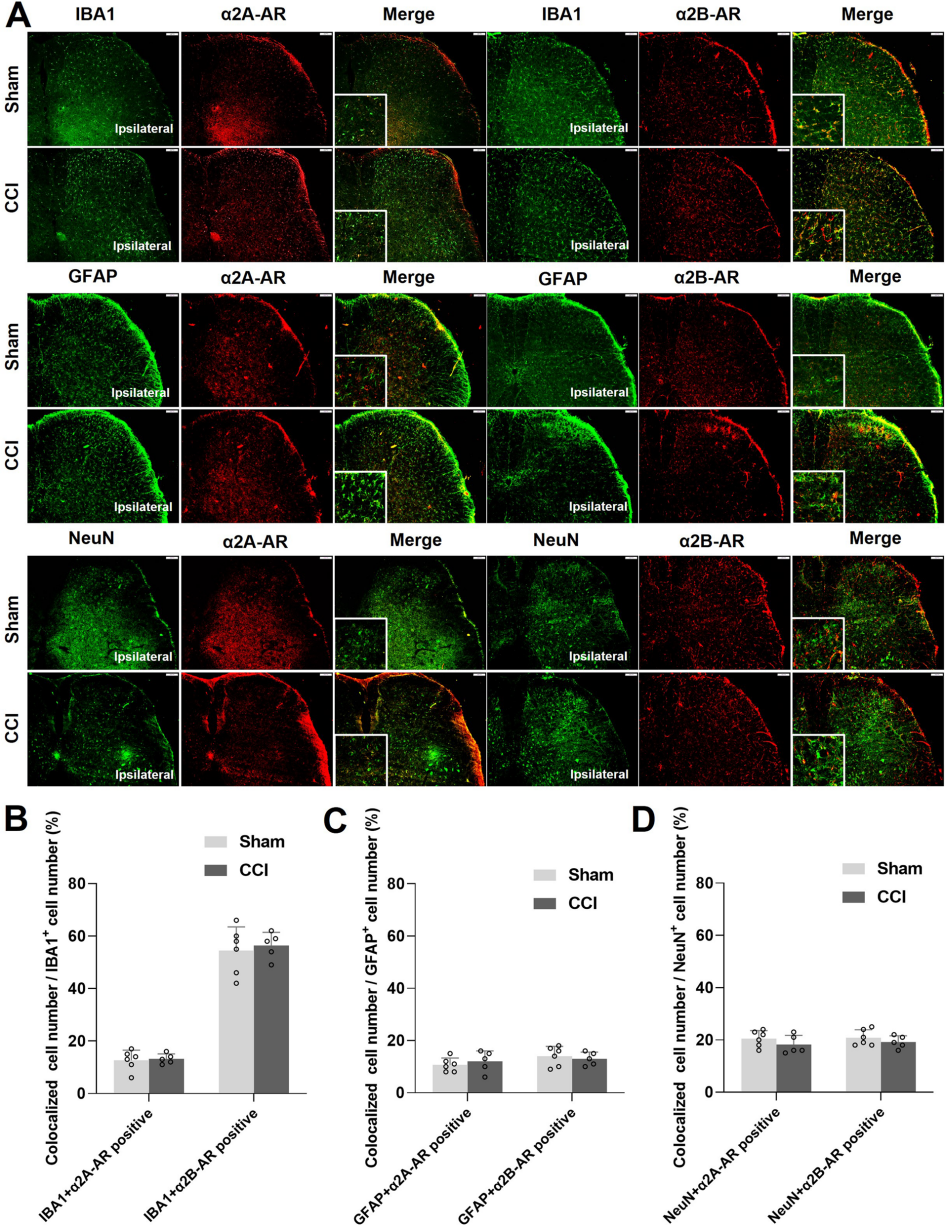
α2-AR expression in SDH. α2A-AR and α2B-AR expression in microglia, astrocyte, and neurons are evaluated with immunofluorescence in mice one week after CCI or sham surgery. **A** Colocalization of α2A-AR and α2B-AR with microglia, astrocyte, and neuron marker, IBA1, GFAP, and NeuN, respectively, in the SDH. **B**–**D** Statistics of the proportion of cell number of double staining (yellow color) cells in the total cells (green). Scale bars = 100 μm. Data are expressed as mean + SEM, *n* = 5–6, 5 slices for each mouse

Because yohimbine can reverse the analgesic effects of LC activation and inhibit neuroinflammation, in order to further evaluate which cells and which α2AR NE acts on, by using immunofluorescence, we observe the expression of α2A-AR and α2B-AR in the spinal cord (L3-5) in CCI mice. The results show that α2A-AR was low expressed in microglia (colocalization < 20%, Fig. 9A, B), low expressed in astrocytes (colocalization < 20%, Fig. 9A, C), and slightly higher in neurons (colocalization ~ 20%, Fig. 9A, D). In addition, α2B-AR is highly expressed in microglia (colocalization > 50%, Fig. 9A, B), low in astrocytes (colocalization < 20%, Fig. 9A, C), and slightly higher in neurons (colocalization ~ 20%, Fig. 9A, D).

## Discussion

To explore the analgesic effect of the LC:SC on NP and its effects on the activation of glial cells and neuroinflammation in SDH, we used DREADD in the present study to selectively activate the LC:SC noradrenergic pathway. The results showed that LC:SC activation increased the release of NE in the SDH and increased pain threshold in CCI mice. In addition, activation of LC:SC for a week suppressed astrocytes and microglia activation, as well as neuroinflammation. These indicate that in NP mice, the analgesic effect of LC:SC activation is partially contributed by inhibiting the neuroinflammation of microglia and astrocytes in SDH. Descending noradrenergic pathways exert a prominent role under the condition of persistent pain, producing an inhibitory effect on pain transmission in inflammatory and NP models [38–40]. The descending pathways from the LC to the spinal cord are activated during peripheral inflammation, and the activation of LC:SC prevents the development of hyperalgesia [41]. Unilateral hind paw inflammation produces the excitation of descending NE-containing neurons from the LC, resulting in an increase of the NE level in the dorsal horn ipsilateral to the site of inflammation [42].

In the present study, the mechanical pain threshold and thermal nociceptive thresholds of CCI mice dropped to the lowest level on the 7th day and maintained after surgery. After intraperitoneal injection of CNO, the LC:SC activated, as evidenced by the increased spike of LC neuron and NE level in SDH. Meanwhile, the CCI mice showed increased pain thresholds and less WDR neuron firing after the activation of LC:SC, which can be erased by α2 receptor blocker yohimbine, suggesting that LC:SC activation can reduce pain intensity of NP mice, which can be contributed by NE and α2 receptor in the SDH. Hughes et al. [43] found that in tibial nerve transection rat, intrathecal yohimbine (30 μg) caused earlier ipsilateral sensitization of all modalities. However, by day 21, established allodynia was not affected by yohimbine. Considering that intrathecal administration of yohimbine may affect the pain threshold of CCI mice, we observed the effect of intrathecal yohimbine on the pain threshold in CCI mice. Intrathecal administration of yohimbine showed no effect on pain threshold and LC activity in mice 2 weeks after CCI (Additional file 1: Fig. S2). This finding is consistent with the report of Hughes et al. [43]. We consider that this may be related to the time of neuropathic pain. Prolonged pain duration (e.g., 2 weeks in this study and 3 weeks in that of Hughes et al.) may cause LC and LC:SC exhaustion [44], resulting in inactivation of the descending analgesic system. However, in the present study, activation of LC with Gq-DREADD had an analgesic effect 2 weeks after CCI, indicating that the resting LC:SC pathway can be activated to relieve chronic pain. Two studies reported that activation of LC:SC was analgesic in NP rat. In the tibial nerve transection NP rat, activating the LC:SC can relieve NP and induce the conditional position preference [6]. Wei et al. found that injection of histamine in LC activated LC:SC and eased NP in rat, although this kind of LC activation was not specific to LC:SC [45]. In another study, the NE reuptake inhibitor, LPM580098, reduced the firing frequency of WDR neurons in the SDH by increasing the content of NE in the SDH to reduce NP in rat [46]. In summary, it can be concluded that activation of the LC:SC inhibits WDR discharge and the transmission of pain.

To observe the effects of long-term LC:SC activation, CNO was given daily for a week. Three days after the last CNO administration, we found that the pain intensity of NP mice was significantly lower than that of the control group, and yohimbine could offset the protective effect, indicating that LC:SC activation for a longer time (one week) has a therapeutic effect on NP, which was partially contributed by α2 receptor. Chronic neuropathic pain can induce neuroplasticity changes in the brainstem adrenergic pathways and spinal α2‐adrenoceptors. After nerve injury, several studies have indicated the increased efficacy of G protein-coupled α2-adrenoceptors as well as potency of α2-adrenoceptor agonists [47, 48]. Studies have revealed an upregulation of spinal α2-adrenoceptors and increased spinal NE content in neuropathic pain. Therefore, nerve injury-induced plasticity in the spinal α2-adrenoceptors represents enhanced descending inhibition or sensitivity of the dorsal horn nociceptive neurons [49, 50]. However, some studies show that activation of α1A-ARs via descending LC noradrenergic signals may be a common mechanism underlying astrocytic Ca^2+^ responses in the SDH evoked by noxious stimuli [51]. Intrathecal NE induces mechanical pain hypersensitivity via α1A-ARs in astrocytes, and chemogenetic stimulation of SDH astrocytes is sufficient to produce the hypersensitivity [16].

In order to explore the mechanism of LC:SC activation in the treatment of NP, we investigated the therapeutic effect of increased content of NE in NP. In addition, considering that central sensitization, which was related to SDH microglia and astrocytes [52], contributes to chronic pain including NP, we hypothesized that NE acted on SDH microglia and astrocytes, inhibited their activation, and alleviated neuroinflammation. When the activation of SDH microglia and astrocytes, and the cytokine content in SDH were detected, we found that the activation of astrocytes and microglia in the SDH of CCI mice decreased after LC:SC activation. Based on this, it is possible that NE acts on glial cells to reduce their activation. Cytokine data indicate that LC:SC activation reduced the production of TNF-α and IL-1β in the SDH of CCI mice. In addition, LC:SC activation reduced the expression of pro-inflammatory cytokines, such as IL-6, iNOS, CD11b, and CD68, and increased the anti-inflammatory cytokines, such as Arg1, IL-4, IL-10, and CD206. Selective elimination of NE fibers in the spinal cord resulted in the increase of activation biomarkers of microglia and astrocytes, further confirming that the anti-inflammatory effects of NE are related to pain relief [53].

In order to determine whether microglia and astrocyte was the source of inflammatory cytokines in SDH, we carried out immunofluorescence co-labeling. We found that microglia produce IL-1β and TNF-α, which can be suppressed by LC:SC activation. It is suggested that LC:SC activation reduces the production of pro-inflammatory cytokines by inhibiting glial cell activation. It is widely reported that occurrence of NP is often accompanied by the activation of glial cells, and the activated glial cells can release various pro-inflammatory cytokines (such as TNF-α, IL-1β, and IL-6), anti-inflammatory cytokines (such as IL-4, Arg1, and IL-10), and various analgesic and pain-causing substances [54, 55]. The pro-inflammatory glial mediators regulate excitatory and inhibitory synaptic transmission in the spinal cord by enhancing nociceptive neurotransmission, which ultimately leads to central sensitization [56, 57]. In the present study, one week of LC:SC activation relieved NP, which can be contributed by the inhibition of overreaction of microglia and astrocytes, and the overexpression of inflammatory cytokine in NP SDH.

Although we observed the increase of NE content in SDH and the decrease of microglia and astrocytes activation after LC:SC activation in NP mice, it was not proved that NE acted directly on SDH glial cells to inhibit their activation. We further observed the expression of α2AR (i.e., α2A-AR and α2B-AR) in SDH. Interestingly, only α2B-AR was highly expressed in microglia in sham and CCI mice, compared with that in astrocyte and neurons. This finding indicates that NE may act directly on α2B-AR in microglia, inhibit their activation, and reduce the microglial neuroinflammation. Other studies had reported that NE acted on α2AR on glial cells and inhibit glial cell activation and neuroinflammation. For example, one study was reported that dexmedetomidine can act on α2AR on spinal microglia to relieve pain in CCI rats [58], suggesting that microglial α2AR can be activated to relieve NP. It was also reported that activation of α2AR can reduce the activation of glial cells after peripheral nerve injury or during chronic inflammation [59]. Therefore, in this study, NE may act on α2AR in glial cells to inhibit the release of inflammatory cytokine, or it may activate the α2AR of WDR neurons to inhibit their excitatory afferents to relieve pain [60].

## Conclusion

In summary, the NP alleviating effects of LC:SC noradrenergic pathway activation may be related to the inhibition of neuroinflammation in microglia and astrocytes in SDH.

## Supplementary Information

**Additional file1. Figure S1.** CNO or CNO + Yohimbine does not affect pain thresholds of the contralateral hind paw in CCI mice. **Figure S2. **Yohimbine does not affect pain thresholds in CCI mice. **Figure S3.** Activation of LC:SC for a week suppresses the activation of microglia and astrocyte in SDH. **Figure S4.** Activation of LC:SC promotes anti-inflammatory cytokines and inhibits pro-inflammatory cytokines in SDH. **Figure S5.** Co-expression of TNF-α, IL-1β with IBA1 in the SDH. **Figure S6. **Co-expression of TNF-α, IL-1β with GFAP in the SDH.

## Data Availability

Please contact the author for data requests.

## Abbreviations

AAV: Adeno-associated virus
CCI: Chronic constriction injury
CNO: Clozapine N-oxide
DREADD: Designer receptors exclusively activated by designer drugs
ELISA: Enzyme-linked immunosorbent assay
LC: Locus coeruleus
NE: Norepinephrine
NP: Neuropathic pain
PBS: Phosphate buffer saline
PFA: Paraformaldehyde
ROI: Regions of interest
SDH: Spinal dorsal horn
TH: Tyrosine hydroxylase
WDR: Wide dynamic range

## References

[1] Bouhassira D (2019). Neuropathic pain: definition, assessment and epidemiology. Rev Neurol (Paris).

[2] Finnerup NB, Haroutounian S, Kamerman P, Baron R, Bennett DLH, Bouhassira D, Cruccu G, Freeman R, Hansson P, Nurmikko T (2016). Neuropathic pain: an updated grading system for research and clinical practice. Pain.

[3] Cao S, Li J, Yuan J, Zhang D, Yu T (2020). Fast localization and sectioning of mouse locus coeruleus. Biomed Res Int.

[4] Pertovaara A (2006). Noradrenergic pain modulation. Prog Neurobiol.

[5] Llorca-Torralba M, Borges G, Neto F, Mico JA, Berrocoso E (2016). Noradrenergic locus coeruleus pathways in pain modulation. Neuroscience.

[6] Hirschberg S, Li Y, Randall A, Kremer EJ, Pickering AE (2017). Functional dichotomy in spinal- vs prefrontal-projecting locus coeruleus modules splits descending noradrenergic analgesia from ascending aversion and anxiety in rats. Elife.

[7] Hayashida KI, Eisenach JC (2018). Descending noradrenergic inhibition: an important mechanism of gabapentin analgesia in neuropathic pain. Adv Exp Med Biol.

[8] Hayashida KI, Kimuram M, Eisenach JC (2018). Blockade of α2-adrenergic or metabotropic glutamate receptors induces glutamate release in the locus coeruleus to activate descending inhibition in rats with chronic neuropathic hypersensitivity. Neurosci Lett.

[9] Di Cesare ML, Micheli L, Crocetti L, Giovannoni MP, Vergelli C, Ghelardini C (2017). α2 adrenoceptor: a target for neuropathic pain treatment. Mini Rev Med Chem.

[10] Seibt F, Schlichter R (2015). Noradrenaline-mediated facilitation of inhibitory synaptic transmission in the dorsal horn of the rat spinal cord involves interlaminar communications. Eur J Neurosci.

[11] Pluvinage JV, Haney MS, Smith BAH, Sun J, Iram T, Bonanno L, Li L, Lee DP, Morgens DW, Yang AC (2019). CD22 blockade restores homeostatic microglial phagocytosis in ageing brains. Nature.

[12] Nieman AN, Li G, Zahn NM, Mian MY, Mikulsky BN, Hoffman DA, Wilcox TM, Kehoe AS, Luecke IW, Poe MM (2020). Targeting nitric oxide production in microglia with novel imidazodiazepines for nonsedative pain treatment. ACS Chem Neurosci.

[13] Morioka N, Tanabe H, Inoue A, Dohi T, Nakata Y (2009). Noradrenaline reduces the ATP-stimulated phosphorylation of p38 MAP kinase via beta-adrenergic receptors-cAMP-protein kinase A-dependent mechanism in cultured rat spinal microglia. Neurochem Int.

[14] Caraci F, Merlo S, Drago F, Caruso G, Parenti C, Sortino MA (2019). Rescue of noradrenergic system as a novel pharmacological strategy in the treatment of chronic pain: focus on microglia activation. Front Pharmacol.

[15] Hinojosa AE, Caso JR, García-Bueno B, Leza JC, Madrigal JL (2013). Dual effects of noradrenaline on astroglial production of chemokines and pro-inflammatory mediators. J Neuroinflammation.

[16] Kohro Y, Matsuda T, Yoshihara K, Kohno K, Koga K, Katsuragi R, Oka T, Tashima R, Muneta S, Yamane T (2020). Spinal astrocytes in superficial laminae gate brainstem descending control of mechanosensory hypersensitivity. Nat Neurosci.

[17] Cao S, Fisher DW, Yu T, Dong H (2019). The link between chronic pain and Alzheimer's disease. J Neuroinflammation.

[18] Tsuda M (2019). Microglia-mediated regulation of neuropathic pain: molecular and cellular mechanisms. Biol Pharm Bull.

[19] Sideris-Lampretsas G, Malcangio M (2021). Microglial heterogeneity in chronic pain. Brain Behav Immun.

[20] Xiong XY, Liu L, Yang QW (2016). Functions and mechanisms of microglia/macrophages in neuroinflammation and neurogenesis after stroke. Prog Neurobiol.

[21] Linnerbauer M, Wheeler MA, Quintana FJ (2020). Astrocyte crosstalk in CNS inflammation. Neuron.

[22] Cao S, Fisher DW, Rodriguez G, Yu T, Dong H (2021). Comparisons of neuroinflammation, microglial activation, and degeneration of the locus coeruleus-norepinephrine system in APP/PS1 and aging mice. J Neuroinflammation.

[23] National Research Council (US) Committee for the Update of the Guide for the Care and Use of Laboratory Animals. Guide for the Care and Use of Laboratory Animals. 8th ed. Washington (DC): National Academies Press (US); 2011. 21595115

[24] Cao S, Deng W, Li Y, Qin B, Zhang L, Yu S, Xie P, Xiao Z, Yu T (2017). Chronic constriction injury of sciatic nerve changes circular RNA expression in rat spinal dorsal horn. J Pain Res.

[25] Tervo DG, Hwang BY, Viswanathan S, Gaj T, Lavzin M, Ritola KD, Lindo S, Michael S, Kuleshova E, Ojala D (2016). A Designer AAV variant permits efficient retrograde access to projection neurons. Neuron.

[26] Zhang Q, Hu DX, He F, Li CY, Qi GJ, Cai HW, Li TX, Ming J, Zhang P, Chen XQ, Tian B (2019). Locus coeruleus-CA1 projections are involved in chronic depressive stress-induced hippocampal vulnerability to transient global ischaemia. Nat Commun.

[27] François A, Low SA, Sypek EI, Christensen AJ, Sotoudeh C, Beier KT, Ramakrishnan C, Ritola KD, Sharif-Naeini R, Deisseroth K (2017). A brainstem-spinal cord inhibitory circuit for mechanical pain modulation by GABA and enkephalins. Neuron.

[28] Cho C, Michailidis V, Lecker I, Collymore C, Hanwell D, Loka M, Danesh M, Pham C, Urban P, Bonin RP, Martin LJ (2019). Evaluating analgesic efficacy and administration route following craniotomy in mice using the grimace scale. Sci Rep.

[29] Hylden JL, Wilcox GL (1980). Intrathecal morphine in mice: a new technique. Eur J Pharmacol.

[30] Huang Q, Sun ML, Chen Y, Li XY, Wang YX (2017). Concurrent bullatine A enhances morphine antinociception and inhibits morphine antinociceptive tolerance by indirect activation of spinal κ-opioid receptors. J Ethnopharmacol.

[31] Luo YJ, Li YD, Wang L, Yang SR, Yuan XS, Wang J, Cherasse Y, Lazarus M, Chen JF, Qu WM, Huang ZL (2018). Nucleus accumbens controls wakefulness by a subpopulation of neurons expressing dopamine D(1) receptors. Nat Commun.

[32] Ahsan MZ, Zhao MJ, Shoaib RM, Zhang Y, Wang YX (2021). Comparative study of dezocine, pentazocine and tapentadol on antinociception and physical dependence. Life Sci.

[33] Wang H, Huang M, Wang W, Zhang Y, Ma X, Luo L, Xu X, Xu L, Shi H, Xu Y (2021). Microglial TLR4-induced TAK1 phosphorylation and NLRP3 activation mediates neuroinflammation and contributes to chronic morphine-induced antinociceptive tolerance. Pharmacol Res.

[34] Kawaguchi M, Satoh Y, Otsubo Y, Kazama T (2014). Molecular hydrogen attenuates neuropathic pain in mice. PLoS ONE.

[35] Otsubo Y, Satoh Y, Kodama M, Araki Y, Satomoto M, Sakamoto E, Pagès G, Pouysségur J, Endo S, Kazama T (2012). Mechanical allodynia but not thermal hyperalgesia is impaired in mice deficient for ERK2 in the central nervous system. Pain.

[36] Chaplan SR, Bach FW, Pogrel JW, Chung JM, Yaksh TL (1994). Quantitative assessment of tactile allodynia in the rat paw. J Neurosci Methods.

[37] Yuan SB, Ji G, Li B, Andersson T, Neugebauer V, Tang SJ (2015). A Wnt5a signaling pathway in the pathogenesis of HIV-1 gp120-induced pain. Pain.

[38] Caputi FF, Nicora M, Simeone R, Candeletti S, Romualdi P (2019). Tapentadol: an analgesic that differs from classic opioids due to its noradrenergic mechanism of action. Minerva Med.

[39] Hayashida KI, Obata H (2019). Strategies to treat chronic pain and strengthen impaired descending noradrenergic inhibitory system. Int J Mol Sci.

[40] Pertovaara A (2013). The noradrenergic pain regulation system: a potential target for pain therapy. Eur J Pharmacol.

[41] Maeda M, Tsuruoka M, Hayashi B, Nagasawa I, Inoue T (2009). Descending pathways from activated locus coeruleus/subcoeruleus following unilateral hindpaw inflammation in the rat. Brain Res Bull.

[42] Tsuruoka M, Hitoto T, Hiruma Y, Matsui Y (1999). Neurochemical evidence for inflammation-induced activation of the coeruleospinal modulation system in the rat. Brain Res.

[43] Hughes SW, Hickey L, Hulse RP, Lumb BM, Pickering AE (2013). Endogenous analgesic action of the pontospinal noradrenergic system spatially restricts and temporally delays the progression of neuropathic pain following tibial nerve injury. Pain.

[44] Parent AJ, Tétreault P, Roux M, Belleville K, Longpré JM, Beaudet N, Goffaux P, Sarret P (2016). Descending nociceptive inhibition is modulated in a time-dependent manner in a double-hit model of chronic/tonic pain. Neuroscience.

[45] Wei H, Jin CY, Viisanen H, You HJ, Pertovaara A (2014). Histamine in the locus coeruleus promotes descending noradrenergic inhibition of neuropathic hypersensitivity. Pharmacol Res.

[46] Li N, Li C, Han R, Wang Y, Yang M, Wang H, Tian J (2019). LPM580098, a novel triple reuptake inhibitor of serotonin, noradrenaline, and dopamine attenuates neuropathic pain. Front Pharmacol.

[47] Donaldson LF, Beazley-Long N (2016). Alternative RNA splicing: contribution to pain and potential therapeutic strategy. Drug Discov Today.

[48] Oladosu FA, Maixner W, Nackley AG (2015). Alternative splicing of G protein-coupled receptors: relevance to pain management. Mayo Clin Proc.

[49] Coull JA, Beggs S, Boudreau D, Boivin D, Tsuda M, Inoue K, Gravel C, Salter MW, De Koninck Y (2005). BDNF from microglia causes the shift in neuronal anion gradient underlying neuropathic pain. Nature.

[50] Hayashida K, Eisenach JC (2011). A tropomyosine receptor kinase inhibitor blocks spinal neuroplasticity essential for the anti-hypersensitivity effects of gabapentin and clonidine in rats with peripheral nerve injury. J Pain.

[51] Kawanabe R, Yoshihara K, Hatada I, Tsuda M (2021). Activation of spinal dorsal horn astrocytes by noxious stimuli involves descending noradrenergic signaling. Mol Brain.

[52] Chen Z, Doyle TM, Luongo L, Largent-Milnes TM, Giancotti LA, Kolar G, Squillace S, Boccella S, Walker JK, Pendleton A (2019). Sphingosine-1-phosphate receptor 1 activation in astrocytes contributes to neuropathic pain. Proc Natl Acad Sci U S A.

[53] Arora V, Morado-Urbina CE, Aschenbrenner CA, Hayashida K, Wang F, Martin TJ, Eisenach JC, Peters CM (2016). Disruption of spinal noradrenergic activation delays recovery of acute incision-induced hypersensitivity and increases spinal glial activation in the rat. J Pain.

[54] Chen G, Park CK, Xie RG, Berta T, Nedergaard M, Ji RR (2014). Connexin-43 induces chemokine release from spinal cord astrocytes to maintain late-phase neuropathic pain in mice. Brain.

[55] Ma L, Li J, Zhou J, Zhang D, Xiao Z, Yu T, Li Y, Cao S (2021). Intravenous lidocaine alleviates postherpetic neuralgia in rats via regulation of neuroinflammation of microglia and astrocytes. iScience.

[56] Zhang ZJ, Jiang BC, Gao YJ (2017). Chemokines in neuron-glial cell interaction and pathogenesis of neuropathic pain. Cell Mol Life Sci.

[57] Tsuda M (2017). P2 receptors, microglial cytokines and chemokines, and neuropathic pain. J Neurosci Res.

[58] Li SS, Zhang WS, Ji D, Zhou YL, Li H, Yang JL, Xiong YC, Zhang YQ, Xu H (2014). Involvement of spinal microglia and interleukin-18 in the anti-nociceptive effect of dexmedetomidine in rats subjected to CCI. Neurosci Lett.

[59] Xu B, Zhang WS, Yang JL, Lû N, Deng XM, Xu H, Zhang YQ (2010). Evidence for suppression of spinal glial activation by dexmedetomidine in a rat model of monoarthritis. Clin Exp Pharmacol Physiol.

[60] Choi S, Yamada A, Kim W, Kim SK, Furue H (2017). Noradrenergic inhibition of spinal hyperexcitation elicited by cutaneous cold stimuli in rats with oxaliplatin-induced allodynia: electrophysiological and behavioral assessments. J Physiol Sci.

