# Supplementary material for: Activation of locus coeruleus-spinal cord noradrenergic neurons alleviates neuropathic pain in mice via reducing neuroinflammation from astrocytes and microglia in spinal dorsal horn
Source: J Neuroinflammation. 2022 May 27;19:123. doi: 10.1186/s12974-022-02489-9 (PMC9145151; doi:10.1186/s12974-022-02489-9)
Supplement: Supplementary file 1 — Additional file1. Figure S1. CNO or CNO + Yohimbine does not affect pain thresholds of the contralateral hind paw in CCI mice. Figure S2. Yohimbine does not affect pain thresholds in CCI mice. Figure S3. Activation of LC:SC for a week suppresses the activation of microglia and astrocyte in SDH. Figure S4. Activation of LC:SC promotes anti-inflammatory cytokines and inhibits pro-inflammatory cytokines in SDH. Figure S5. Co-expression of TNF-α, IL-1β with IBA1 in the SDH. Figure S6. Co-expression of TNF-α, IL-1β with GFAP in the SDH. [file 12974_2022_2489_MOESM1_ESM.docx]

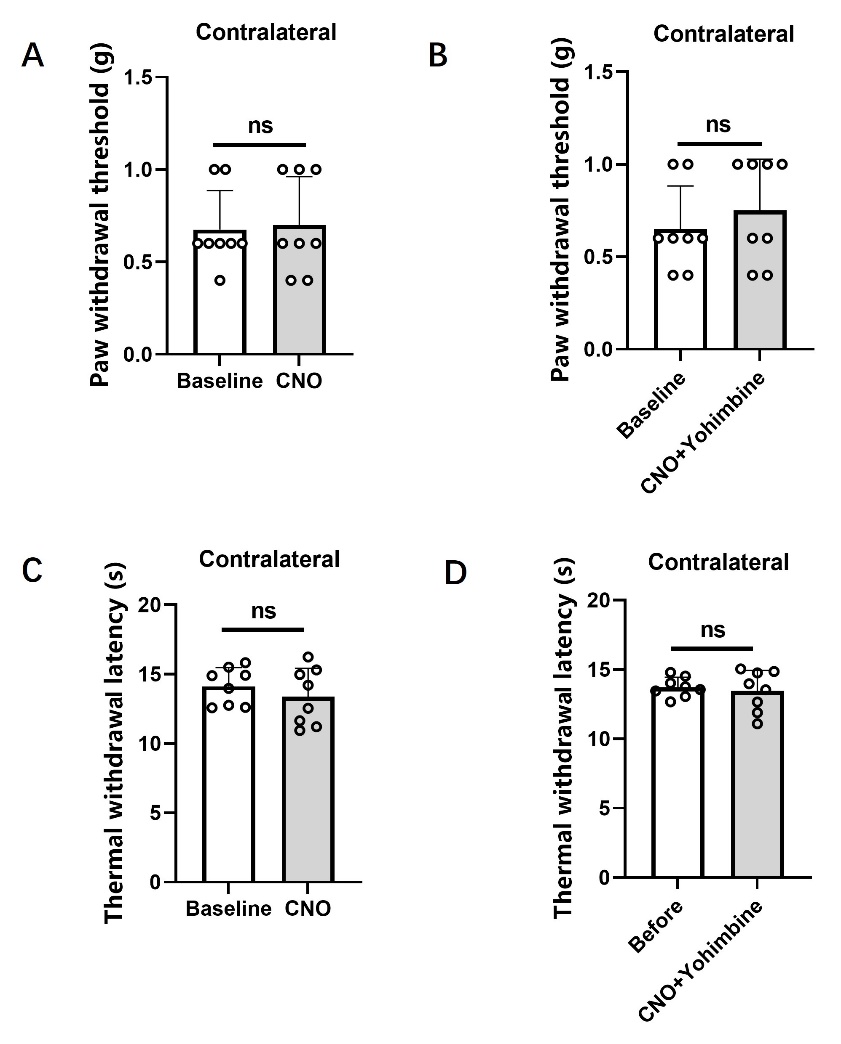


**Figure S1. CNO or CNO+Yohimbine does not affect pain thresholds of the contralateral hind paw in CCI mice.**

**(A-B)** Paw withdrawal thresholds are tested with von Frey filaments. **(C-D)** Thermal withdrawal latencies are measured by a plantar thermal testing apparatus. Data are expressed as mean + SEM, n=8 for mechanical withdrawal threshold tests and thermal withdrawal latency tests. ns: not statistically significant.


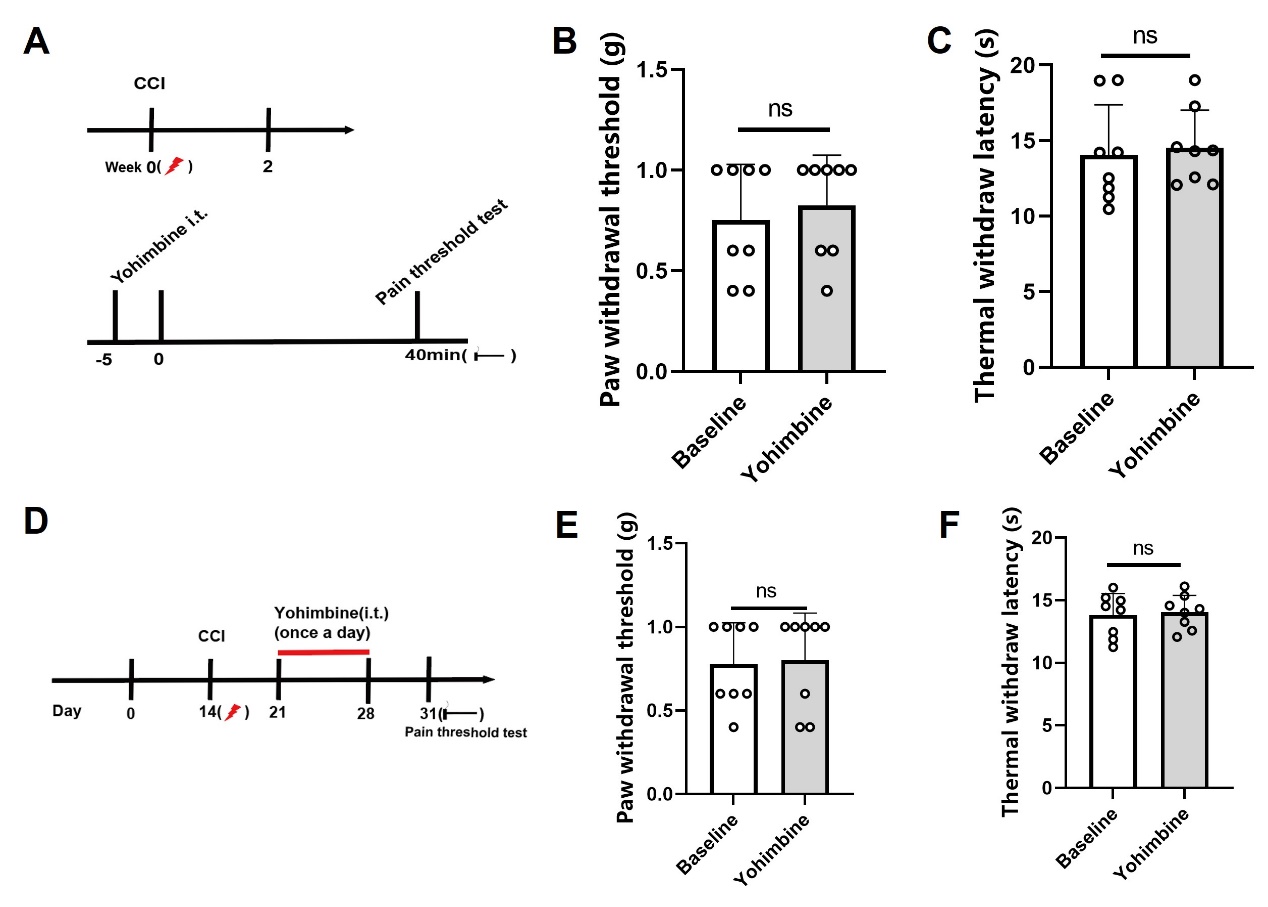


**Figure S2. Yohimbine does not affect pain thresholds in CCI mice.**

Paw withdrawal thresholds are tested with von Frey filaments. Thermal withdrawal latencies are measured by a plantar thermal testing apparatus. **(A)** Experimental protocol for pain threshold test. i.t.: intrathecal injection. **(B-C)** Intrathecal injection of yohimbine do not affect the pain threshold of mice 2 weeks after CCI. **(D)** Experimental protocol for CCI surgery and pain threshold test. i.t.: intrathecal injection. **(E-F)** Intrathecal injection of yohimbine do not affect the pain threshold of mice 17 days after CCI. Data are expressed as mean + SEM, n=8 for mechanical withdrawal threshold tests and thermal withdrawal latency tests. ns: not statistically significant.


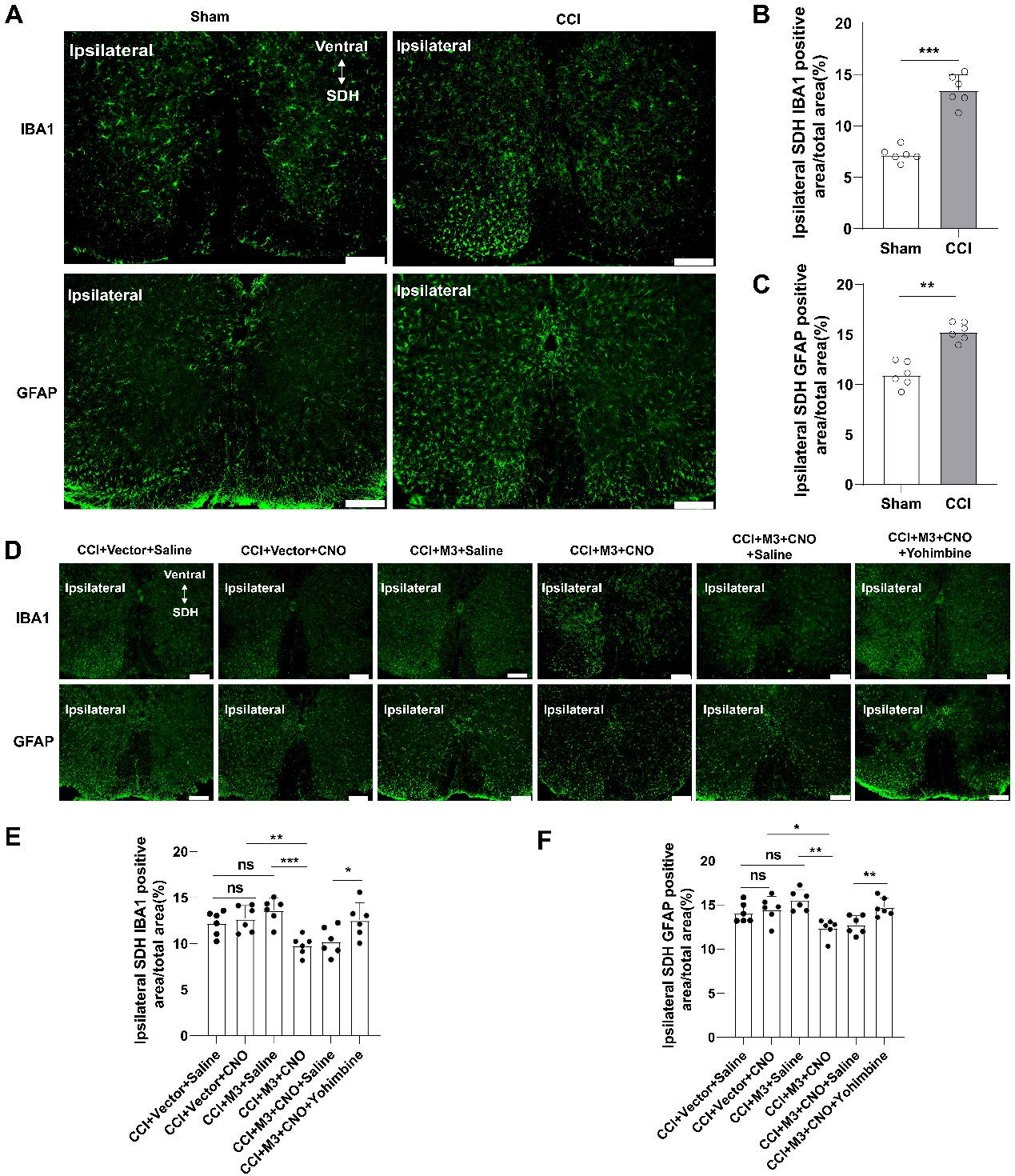


**Figure S3. Activation of LC:SC for a week suppresses the activation of microglia and astrocyte in SDH. (A)** Immunofluorescence images of microglia and astrocytes in the SDH in CCI mice. Scale bars=100 μm. **(B-C)** Proportions of IBA1 positive area **(B)** and GFAP positive area **(C)** in the SDH, which indicated that microglia and astrocyte was activated after CCI surgery. **(D)** Activation of LC:SC suppresses the activation of microglia and astrocyte in SDH. Scale bars=100 μm. **(E-F)** Statistics of the proportion of IBA1 **(E)** and GFAP **(F)** positive area in the SDH, which indicated that one week of LC:SC activation can reverse the activation of astrocyte and microglia in CCI mice. Data were expressed as mean+SEM, n=6, 5 slices for each mouse. ^*^p<0.05, ^**^p<0.01, ^***^p<0.001. ns=not statistically significant.


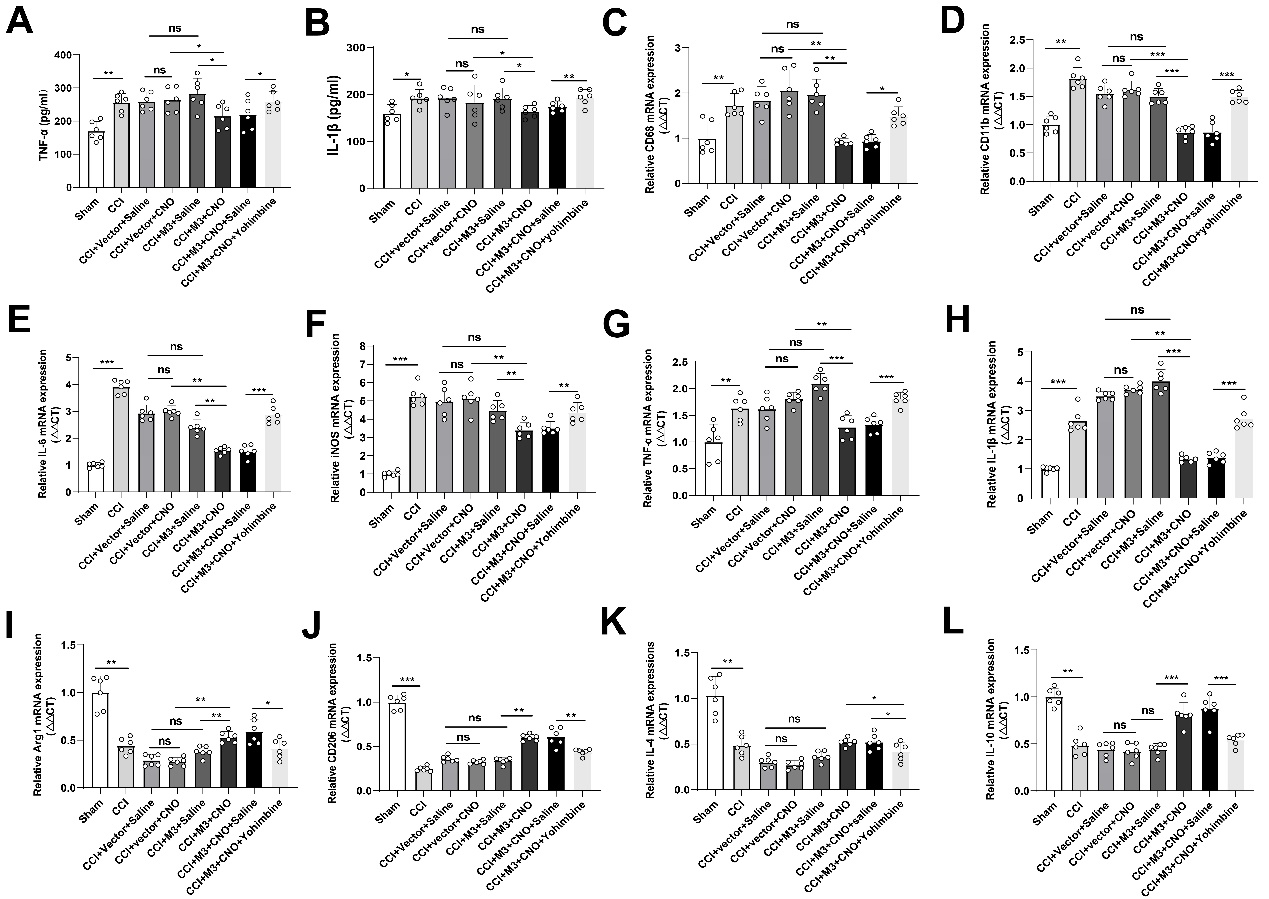


**Figure S4. Activation of LC:SC promotes anti-inflammatory cytokines and inhibits pro-inflammatory cytokines in SDH. (A)** CCI increased TNF-α expression in SDH, whereas activation of LC:SC inhibited the expression of TNF-α. **(B)** CCI increased IL-1β expression in SDH, whereas activation of LC:SC inhibited the expression of IL-1β. **(C-H)** CCI increased the mRNA expression of TNF-α, IL-1β, CD68, IL-6, iNOS and CD11b, whereas activation of LC:SC inhibited the expression of these mRNAs in the SDH of CCI mice. Yohimbine reversed LC:SC activation induced down-regulation of these pro-inflammation mRNAs. **(I-L)** CCI inhibited the mRNA expression of Arg1, CD206, IL-4, and IL-10, whereas activation of LC:SC increased the expression of these mRNAs in the SDH of CCI mice. Yohimbine reversed LC:SC induced up-regulation of these anti-inflammation mRNAs. Data were expressed as mean+SEM, n=6. Statistical analyses consisted of one-way ANOVA tests followed by Tukey’s post-hoc tests. ^*^p<0.05, ^**^p<0.01, ^***^p<0.001. ns=not statistically significant.


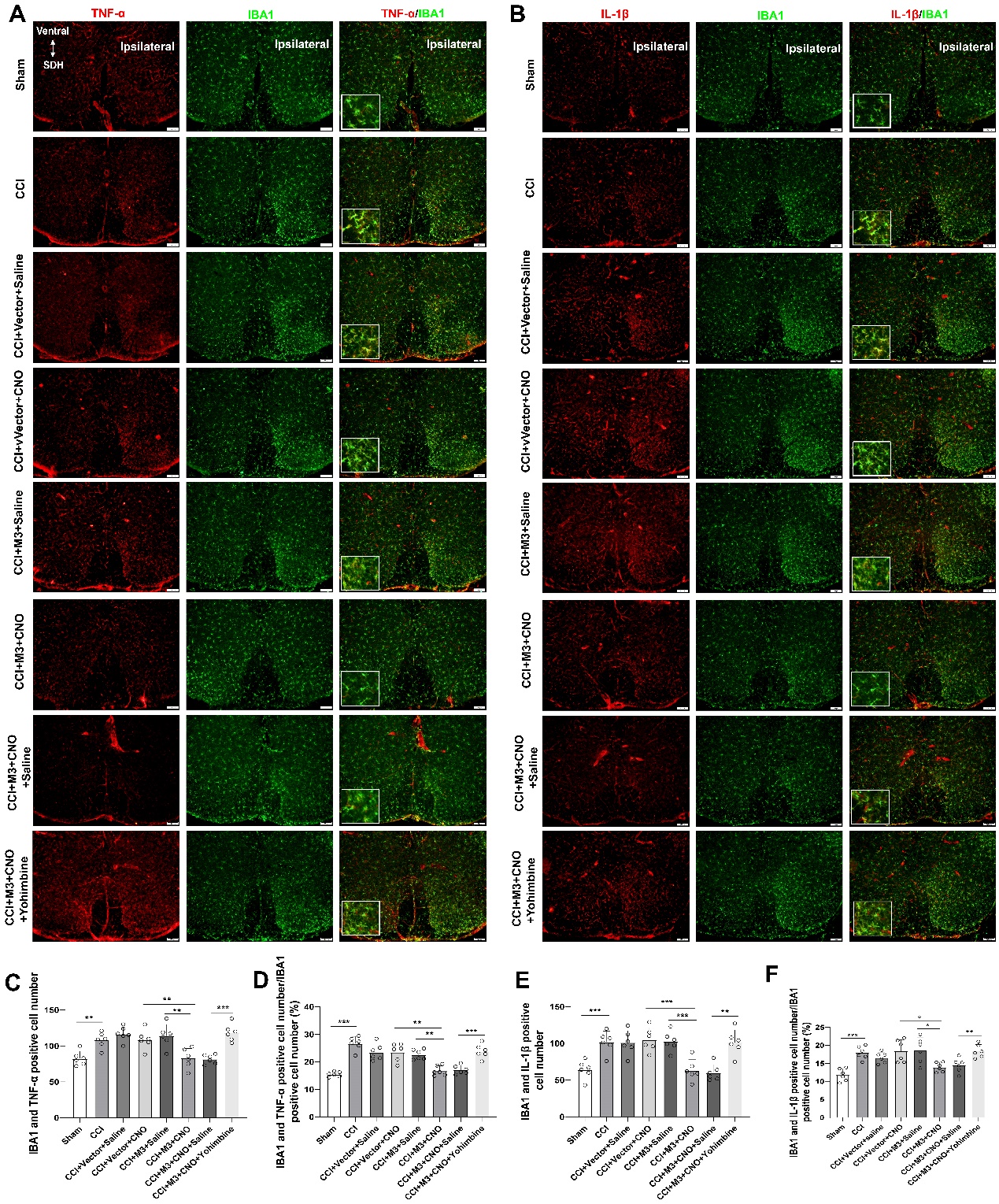


**Figure S5. Co-expression of TNF-α, IL-1β with IBA1 in the SDH. (A)** Colocalization of TNF-α (red) and IBA1 (green) in the SDH following CCI. More co-localized microglia (yellow) can be seen in mice with CCI. **(B)** The representative images of double immunofluorescence staining showing that IL-1β (red) colocalized with IBA1 (green) in SDH. **(C-D)** Shown in relative number and in percentage, colocalization of TNF-α and IBA1 in CCI group increased compared to the Sham group. Compared with the CCI+M3+Saline group, colocalization of TNF-α and IBA1 of the CCI+M3+CNO group is reduced and can be reversed by yohimbine. **(E-F)** Colocalization of IL-1β and IBA1 in CCI group increased compared to the Sham group. Compared with the CCI+M3+Saline group, colocalization of IL-1β and IBA1 of the CCI+M3+CNO group is reduced and can be reversed by yohimbine. Scale bars=100 μm. Data were expressed as mean+SEM, n=6, 5 slices for each mouse. ^*^p<0.05, ^**^p<0.01, ^***^p<0.001.


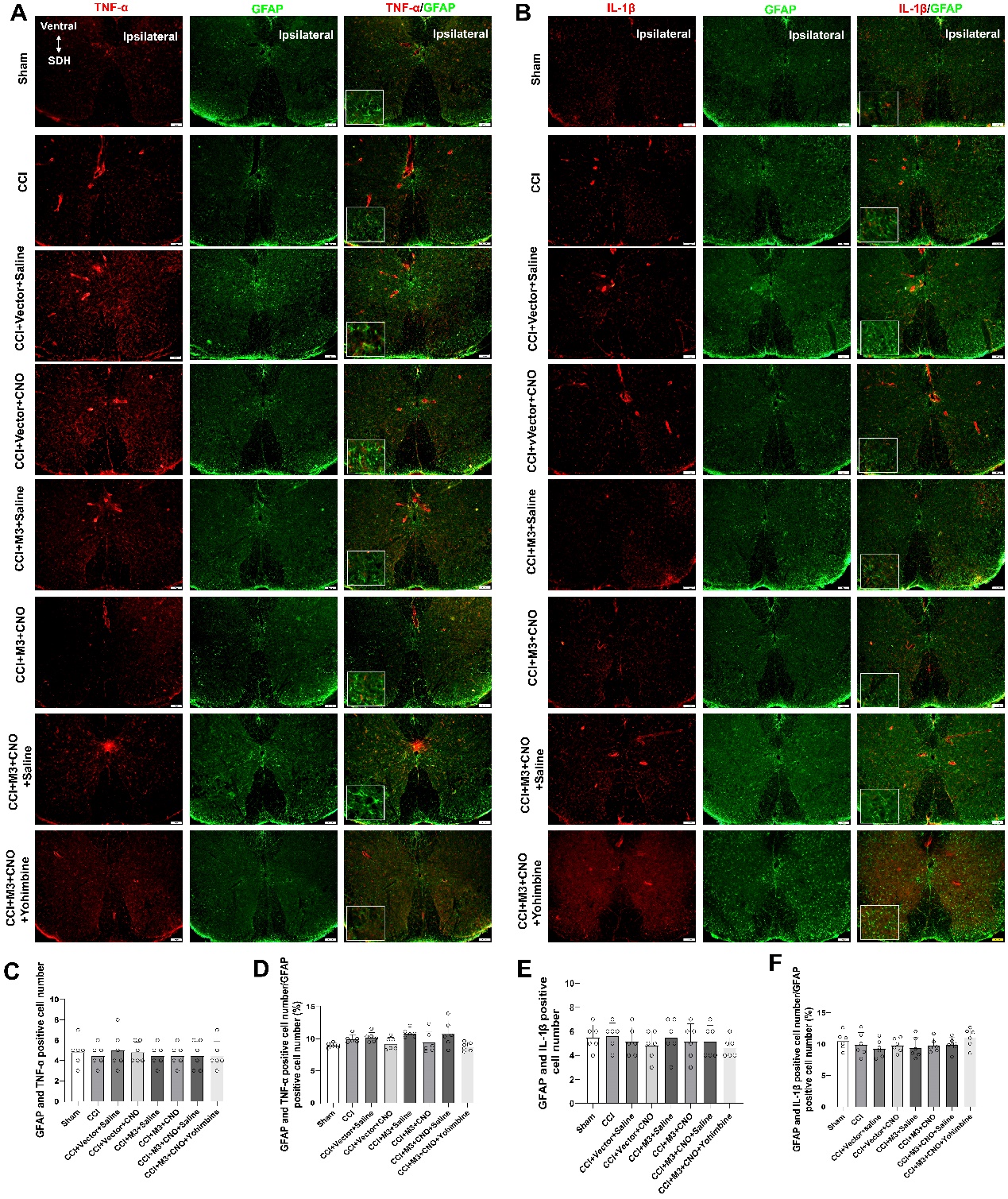


**Figure S6. Co-expression of TNF-α, IL-1β with GFAP in the SDH. (A)** Colocalization of TNF-α (red) and GFAP (green) in the SDH following CCI. Not too much co-localized astrocytes (yellow) can be seen in mice with CCI. **(B)** The representative images of double immunofluorescence staining showing that IL-1β (red) is not well colocalized with GFAP (green) in SDH. **(C-D)** Shown in relative number and in percentage, colocalization of TNF-α and GFAP in groups was comparable. **(E-F)** Shown in relative number and in percentage, colocalization of IL-1β and GFAP in groups was comparable. Scale bars=100 μm. Data were expressed as mean+SEM, n=6, 5 slices for each mouse.
